# Monocyte-Platelet Aggregates Triggered by CD31 Molecule in Non-ST Elevation Myocardial Infarction: Clinical Implications in Plaque Rupture

**DOI:** 10.3389/fcvm.2021.741221

**Published:** 2022-01-25

**Authors:** Ramona Vinci, Daniela Pedicino, Alice Bonanni, Alessia d'Aiello, Eugenia Pisano, Myriana Ponzo, Anna Severino, Pellegrino Ciampi, Francesco Canonico, Giulio Russo, Marianna Di Sario, Rocco Vergallo, Simone Filomia, Rocco Antonio Montone, Davide Flego, Lucia Stefanini, Roberto Piacentini, Cristina Conte, Francesco Cribari, Massimo Massetti, Filippo Crea, Giovanna Liuzzo

**Affiliations:** ^1^Department of Cardiovascular and Pulmonary Sciences, Università Cattolica del Sacro Cuore, Rome, Italy; ^2^Department of Cardiovascular Sciences, Fondazione Policlinico Universitario A. Gemelli IRCCS, Rome, Italy; ^3^Department of Internal Medicine and Medical Specialties, Sapienza University of Rome, Rome, Italy; ^4^Department of Neuroscience, Università Cattolica del Sacro Cuore, Rome, Italy; ^5^Fondazione Policlinico Universitario A. Gemelli IRCCS, Rome, Italy

**Keywords:** acute coronary syndromes, thrombus burden, unstable plaque, plaque rupture, monocyte-platelet aggregates, CD31, precision medicine

## Abstract

Despite the recent innovations in cardiovascular care, atherothrombosis is still a major complication of acute coronary syndromes (ACS). We evaluated the involvement of the CD31 molecule in thrombotic risk through the formation of monocyte-platelet (Mo-Plt) aggregates in patients with ACS with no-ST-segment elevation myocardial infarction (NSTEMI) on top of dual anti-platelet therapy (DAPT). We enrolled 19 control (CTRL) subjects, 46 stable angina (SA), and 86 patients with NSTEMI, of which, 16 with Intact Fibrous Cap (IFC) and 19 with Ruptured Fibrous Cap (RFC) as assessed by the Optical Coherence Tomography (OCT). The expression of CD31 on monocytes and platelets was measured. Following the coronary angiography, 52 NSTEMIs were further stratified according to thrombus grade (TG) evaluation. Finally, a series of *ex vivo* experiments verified whether the CD31 participates in Mo-Plt aggregate formation. In patients with NSTEMI, CD31 was reduced on monocytes and was increased on platelets, especially in NSTEMI presented with RFC plaques compared to those with IFC lesions, and in patients with high TG compared to those with zero/low TG. *Ex vivo* experiments documented an increase in Mo-Plt aggregates among NSTEMI, which significantly decreased after the CD31 ligation, particularly in patients with RFC plaques. In NSTEMI, CD31 participates in Mo-Plt aggregate formation in spite of optimal therapy and DAPT, suggesting the existence of alternative thrombotic pathways, as predominantly displayed in patients with RFC.

## Introduction

Despite the advances in medical treatments and interventional innovations, the prevalence of acute coronary syndromes (ACS) is still high (1). Anti-thrombotic drugs represent the first-line of therapeutic choice; however, recurrences and bleeding risk (2) remain two of the main concerns in the management of the patient (3–7).

The rupture of a lipid-rich plaque, with the consequent release of highly thrombotic elements, characterizes at least 50% of patients with ACS (8). Furthermore, atherosclerotic lesions with superimposed thrombus show increased levels of platelet-leucocyte aggregates that could contribute to the generation of further adverse events (9–11).

Each plaque phenotype may be associated with a different thrombotic burden and this may be due to alternative pathogenic mechanisms (12–17). Intracoronary imaging using optical coherence tomography (OCT) allows us to distinguish between a ruptured fibrous cap (RFC) and an intact fibrous cap (IFC) lesion. Histological analysis of thrombus aspirates from patients with ACS revealed a reduced thrombotic burden in patients with IFC, compared with those with RFC, which was enriched by inflammatory infiltrates (18).

The platelet endothelial cell adhesion molecule CD31 is a transmembrane immunoglobulin-like glycoprotein of about 130 kilodaltons (kDa) expressed on the surface of leukocytes, endothelial cells, and platelets (19–21); it exerts multiple functions using six Ig-like domains, building homophilic and/or heterophilic bindings (22–24), although mechanisms under these interactions are mostly unknown. Aside from these Ig-like extracellular domains, CD31 consists of a transmembrane region and a cytoplasmatic tail, whereby the molecule triggers co-inhibitory, pro-survival, and downstream pathways (25). The role of CD31 is recognized in immune cell transmigration; indeed, cellular trafficking across vascular endothelium can be inhibited, both *in vitro* and *in vivo*, by anti-CD31 antibodies (26, 27).

Alongside, CD31 carries out an immunomodulatory role limiting the activation of T- and B-cells (28–31), as well as it exerts a regulatory role on platelet activation (32).

In patients presenting with ACS, the reduction of CD31 in leukocytes is associated with increased inflammation (33).

Furthermore, exposure to CD31 at the site of the vascular injury could amplify platelet adhesion and could lead to the formation of plugs. This could promote a further expansion of inflammatory signaling by initiating a homing activity through the recruitment of other cells expressing CD31 on their surface, such as monocytes and granulocytes (19).

However, the contribution of CD31 in platelet activation is largely unknown and requires further evaluation. Few results have shown that the antibodies directed against CD31 can reduce platelet aggregation (34–36), suggesting a role of this molecule in platelet aggregation and thrombus formation.

This study investigates the following: (1) the expression of CD31 on monocytes and platelets in ACS patients, patients with stable angina (SA), and control (CTRL) subjects; (2) the different role of CD31 in patients with ACS presented with RFC and IFC at the site of the culprit stenosis, according to OCT assessment; and (3) the involvement of CD31 in monocyte-platelet cross-talk and the coronary thrombus burden severity.

## Materials and Methods

### Study Population

Our population included a total of 151 individuals: (1) 86 patients with Acute Coronary Syndrome (ACS) admitted to our Coronary Care Unit (CCU) with a diagnosis of Non-ST Elevation Myocardial Infarction (NSTEMI) confirmed at coronary angiography (37); (2) 46 patients with Stable Angina (SA) with symptoms of stable effort angina lasting more than 1 year, angiographically confirmed coronary artery disease, with any precedent acute coronary events, and any evident ischemic episodes during the last 48 h (38); (3) 19 Control (CTRL) subjects without apparent clinical sign of coronary artery disease screened during their cardiovascular prevention medical examination. In addition, we analyzed a group of ST-Elevation Myocardial Infarction (STEMI, *n* = 16) patients enrolled in the Cath Lab, at the time of primary percutaneous coronary intervention (refer to Supplementary Table 1 for characteristics of the patient group).

Exclusion criteria were: (1) age <18 or >80 years; (2) severe chronic heart failure, i.e., New York Heart Association (NYHA) functional classes III and IV with Left Ventricular Ejection Fraction (LVEF) <35%; (3) severe heart valve disease; (4) recent (<3 months) major surgical procedures or trauma; (4) in-stent restenosis, stent thrombosis, and culprit lesion in a saphenous vein graft; (3) autoimmune diseases, evidence of immunologic disorders, or chronic infectious disease; (4) liver diseases; (5) use of anti-inflammatory or immunosuppressive drugs other than low-dose aspirin; (6) malignancies; and (7) chronic kidney disease stage 4 (GFR < 30 ml/min).

Clinical features were carefully recorded at the time of patient admission and enrollment. Patients were all matched for age *(p* = 0.826).

The main population characteristics are listed in Table 1. All individuals gave their informed consent. Our local Ethics Committee approved the study (Protocol No 36077/19 ID 2747).

**Table 1:** Baseline characteristics of the study population.

	**CTRL *n =* 19**	**SA *n =* 46**	**NSTEMI *n =* 86**	***P*-value**
Age, mean ± SD	64 ± 9.7	66 ± 9.9	65 ± 13.5	0.826
Gender, M/F	9/10	38/8	58/28	0.016
**CV risk factors**				
Smoking, (%)	3 (16)	26 (57)	43 (50)	0.009
Diabetes, (%)	0 (0)	18 (39)	27 (33)	0.006
Hypertension, (%)	9 (47)	40 (87)	65 (78)	0.002
Dyslipidemia, (%)	4 (21)	30 (65)	45 (54)	0.005
Obesity, (%)	3 (16)	3 (7)	20 (24)	0.042
Family history, (%)	2 (11)	15 (33)	32 (39)	0.064
Recurrence, (%)	–	–	13 (15)	–
**Medical therapy**				
DAPT, (%)^#^	–	17 (37)	40 (47)	0.001
ASA, (%)	2 (14)	37 (80)	55 (65)	<0.001
Clopidogrel, (%)	–	18 (19)	19 (23)	0.03
Prasugrel, (%)	–	0	0	–
Ticagrelor, (%)	–	2 (4)	24 (34)	<0.001
Anticoagulants, (%)	2 (14)	2 (4)	7 (8)	0.455
Beta-Blockers, (%)	7 (37)	29 (63)	40 (47)	0.088
Diuretics, (%)	2 (11)	10 (22)	22 (23)	0.360
ACE-I, (%)	2 (11)	13 (28)	33 (38)	0.051
ARBs, (%)	5 (26)	22 (48)	25 (29)	0.07
Statins, (%)	6 (32)	32 (70)	41 (48)	0.009
Ca-antagonists, (%)	0	12 (26)	15 (17)	0.044
Nitrates, (%)	0	3 (7)	1 (1)	0.140
Insulin, (%)	0	5 (11)	4 (5)	0.179
Oral antidiabetic, (%)	0	11 (24)	19 (22)	0.066
**Laboratory Values, mean** **±SD**				
CK, μg/l	75 ± 19	135 ± 139	169 ± 128	0.273
CKMB, μg/l	1 ± 0.3	4 ± 2.7	25 ± 51	0.768
Tn I, ng/ml	–	0.01 ± 0.1	6.7 ± 12.1	0.017
Hb, g/dl	14.9 ± 0.7	13.3 ± 2.3	13.3 ± 2.1	0.447
WBC, x10^3^/ml	7.2 ± 1.6	9.2 ± 2.1	9.6 ± 2.8	0.326
Platelets, x10^3^/ml	220 ± 28	217 ± 52	244 ± 58	0.030
Lymphocytes, x10^9^/l	2.1 ± 0.7	2.4 ± 1	2.2 ± 1.1	0.846
Lymphocytes, %	29 ± 7	27 ± 9	24 ± 9	0.493
Glycemia, mg/dl	100 ± 24	109 ± 35	120 ± 52	0.318
Creatinine, mg/dl	0.8 ± 0.2	1.0 ± 0.4	0.9 ± 0.5	0.54
Cholesterol, mg/dl	219 ± 50	154 ± 44	164 ± 45	0.02
LDL-cholesterol, mg/dl	129 ± 22	86 ± 37	100 ± 35	0.04
HDL-cholesterol, mg/dl	65 ± 12	44 ± 10	51 ± 11	0.468
Triglycerides, mg/dl	128 ± 37	109 ± 42	139 ± 51	0.030
ESR, mm/h	13 ± 4	12 ± 15	26 ± 25	0.537
hs-CRP, mg/l	2.1 ± 0.7	3.0 ± 4.1	11.9 ± 18.2	0.09

# *These data refer to the time of the enrollment of the patient and blood withdrawal. At the time of coronary angiography, all the patients with NSTEMI were on DAPT according to current guidelines (4). ACE-I, ACE inhibitors; ARBs, angiotensin II receptor blockers; ASA, aspirin; Ca, calcium; CTRL, control individuals; CV, cardiovascular; CK, creatine kinase; DAPT, dual antiplatelet therapy; ESR, erythrocyte sedimentation rate; Hb, hemoglobin; HDL, high-density lipoprotein; hsCRP, high sensitive C-reactive protein; LDL, low-density lipoprotein; M/F, male/female; SA, stable angina; NSTEMI, Non ST-segment elevation myocardial infarction; SD, standard deviation; Tn, troponin; and WBC, white blood cells*.

### Hematological Routine Tests

Venous blood samples were collected at the time of hospital admission for hematological routine tests. Total and differential white blood cell counts were analyzed on fresh blood samples with a Bayer H^*^3-hematology analyzer (Leverkusen, Germany), using automated flow cytochemistry. Serum cardiac troponin I (cTnI) was determined at the time of hospital admission as routine measurement by high-sensitivity Single Molecule Counting technology (ADVIA Centaur immunoassay system, Siemens, Erlangen, Germany Roche Diagnostics, Mannheim, Germany). The minimum detectable concentration was 0.04 ng/ml (99th percentile in healthy individuals). Moreover, high-sensitive-CRP (hs-CRP) was measured using a high-sensitivity latex-enhanced immunonephelometric assay (Latex/BN II, Dade Behring, Marburg, Germany).

### Optical Coherence Tomography (OCT)

Optical coherence tomography (OCT) interrogation was performed before the stent implantation for clinical reasons, by using the OCT system C7-XR (St. Jude Medical, St. Paul, MN). Therefore, not all patients underwent an OCT interrogation. Moreover, for further analysis, we considered only those patients in whom it was technically possible to clearly identify the feature of the culprit plaques. We classified as plaques with Intact Fibrous Cap all lesions with thrombus overlying a plaque characterized by an intact fibrous cap, or with the presence of irregularities of the luminal surface at the culprit site in the absence of thrombus. On the other hand, we classified as Rupture Fibrous Cap (RFC) all the lesions characterized by a discontinuity of the fibrous cap that presents a cavity formed inside the plaque or with direct communication between the lumen and inner core of the lesion (39–41).

Two experienced investigators, who were blinded about the clinical information, performed the OCT analyses using the established criteria (refer to above), with an inter-observer agreement of 86.4% (*K* = 0.7; *p* < 0.001), and with intra-observer reliability of the two investigators between 95% (*K* = 0.8; *p* < 0.0001) and 100% (*K* = 1; *p* < 0.0001). To assess the OCT intra-observer reliability, the investigators reapplied the same criteria for OCT analysis at least 1 month after the first reading. In case of discordance, a consensus was obtained involving a third investigator.

The 35 patients with NSTEMI, who underwent OCT analysis of culprit coronary lesions, were sub-grouped in RFC (*n* = 19) and IFC (*n* = 16), beyond the unknown plaque phenotypes that were excluded (*n* = 5).

Angiographic and OCT findings are shown in Supplementary Table 2.

### Thrombus Grade Evaluation

Angiographic thrombus burden was categorized into 5 grades, as described in a previous study (42, 43), providing high intra- and inter-observer agreements. In particular, thrombus grade 0 (zero) defines the absence of angiographic characteristics of thrombus; thrombus grade 1 defines the possible presence of thrombus (i.e., reduced contrast density, haziness, and irregular lesion contour); thrombus grade 2 corresponds to definite thrombus, with greatest linear dimension ≤ 1/2 vessel diameter; thrombus grade 3 corresponds to definite thrombus, with greatest linear dimension >1/2 but <2 vessel diameters; thrombus grade 4 corresponds to definite thrombus, with greatest linear dimension ≥2 vessel diameters; and thrombus grade 5 corresponds to total coronary occlusion. Lesions with a thrombus grade from 0 to 2 were classified as having a zero/low thrombus burden, while those with a thrombus grade from 3 to 5 were classified as having a high thrombus burden.

Two blinded investigators performed a thrombus grade analysis, with an inter-observer agreement of 80.5% (*K* = 0.7; *p* < 0.0001), and an intra-observer reliability of the two investigators between 87% (*K* = 0.8; *p* < 0.0001) and 94.8% (*K* = 0.9; *p* < 0.0001). To assess the thrombus grade intra-observer reliability, the investigators reapplied the same criteria for thrombus grade analysis at least 1 month after the first reading. In case of discordance, a consensus was obtained involving a third investigator.

### Blood Sampling and Isolation of Human Peripheral Mononuclear Cells

At the time of study enrollment, 30 cc of venous blood samples were collected, within 24 h from the onset of symptoms (9 ± 3 h). Peripheral blood mononuclear cells (PBMCs) were isolated from whole blood EthyleneDiamineTetraacetic Acid (EDTA) samples by density gradient centrifugation method at 1,200 x g for 25 min at room temperature (RT) (with no brake applied) (Lympholyte®-H Cell Separation Media, CEDARLANE, Burlington, Canada). The pellets of PBMC were washed and were resuspended in Dulbecco's phosphate-buffered saline (DPBS) (GIBCO, Invitrogen, Carlsband, CA, USA), and were aliquoted according to the final analyses. Cell concentration was determined by using an automated cell counter (Nucleocounter, ChemoMetec, Allerod, Denmark).

### Isolation of Human Platelets

Whole blood citrate dextrose samples were centrifuged at 200 x g for 15 min at RT (with no brake applied). The top 3/4 of the platelet-rich plasma (PRP) was transferred into a new plastic tube, without disturbing the buffy coat layer, and was centrifuged as above for 5 min to remove by pelleting the residual erythrocytes/white blood cells. The 2/3 of supernatant was withdrawn and centrifuged in Acid-Citrate-Dextrose (ACD) solution (1-part ACD solution to 9 parts blood) (Sigma-Aldrich, S. Louis, MO, USA) at 800 x g for 20 min (with no brake applied). Platelet pellets were gently washed and resuspended in Hepes-Tyrode buffer pH 7.4 and aliquoted according to the final analyses. Before the flow cytometry analyses, to ensure that platelets cannot be induced to a new functional state, we used ThromboFix Platelet stabilizer (Beckman Coulter, Brea, CA); moreover, during all the procedures, strong mechanical forces (i.e., fast pipetting or vigorous shaking) have been avoided, while blood and reagents were always kept and handled at RT or 37°C.

### Flow Cytometry Immunophenotyping and Analysis of Basal Monocytes and Platelets

All the acquisitions on the fresh starting materials were made possible by the close proximity of the clinic and the laboratory areas, which are both situated in the Department of Cardiovascular and Pulmonary Sciences at Fondazione Policlinico Universitario A. Gemelli IRCCS. CD31 expression on basal PBMCs and platelets was assessed by flow cytometry after staining with monoclonal (m) antibody (Ab) anti-CD31-Phycoerytrin (PE) (clone 1F11, IM2409; Beckman Coulter, Brea, CA, USA). We used an anti-CD14-Electron Coupled Dye (ECD) (clone RM052, B92391; Beckman Coulter, Brea, CA, USA) and a CD42b-fluorescein isothiocyanate (FITC) (clone SZ2, IM0648U; Beckman Coulter, Brea, CA, USA) as monocyte and platelet markers, respectively. We used anti-CD45-PE (clone J33, Beckman Coulter, Brea, CA, USA) for verifying the purity of our platelet samples and a mAb anti-CD62 P-selectin (P) (clone Psel.KO2.3, 12-0626-82; eBioscience, INC., San Diego, CA, USA) to determine the platelet activation. All the antibody staining were performed for 15 min at RT and under a dark condition. The expression of each Median Fluorescence Intensity (MFI) was assessed by subtracting the negative peak-MFI from the positive peak-MFI. After the incubation, cells were washed with 0.5 ml of 1X DPBS (Gibco, Thermo-Fisher, Waltham, MA, USA) and were pelleted by centrifugation (250 × g for 5 min); the supernatant was discarded. Finally, cells were resuspended in 1 ml of 1X DPBS (Gibco, Thermo-Fisher, Waltham, MA, USA) for flow cytometry analysis. For each acquisition, a total of 50,000 events were captured. Flow cytometry analyses were conducted with Cytomics FC500 Analyzer (lasers: blue 488 nm, red 631 nm; serial number AH20082) (Beckman Coulter, Brea, CA, USA) and data were analyzed with Kaluza software (Beckman Coulter, Brea, CA, USA) (44) (Supplementary Figures 1, 2).

### *Ex vivo* Co-culture Experiments for Evaluating Monocyte-Platelet Aggregates and Effect of CD31 Ligation

Freshly isolated PBMCs of 2 × 10^6^/ml were co-cultured in 12-well plates with freshly isolated platelets in a ratio of 1:1 for 16 h at 37°C under 5% carbon dioxide (CO_2_) and 20% Oxigen (O_2_), in Roswell Park Memorial Institute (RPMI) 1640 medium (LONZA, Verviers, Belgium) supplemented with 100 U penicillin, 0.1 mg/ml streptomycin, 2 mmol glutamine, and 10% Fetal Bovine Serum (FBS) (Thermo-Fisher, Waltham, MA, USA). The mAb anti-CD31 was added to the culture medium (1 μg/ml, clone WM59, 16-0319-82, Functional Grade; eBioscience, San Diego CA, USA) for CD31 ligation experiments. Furthermore, we performed the above-described experiments in presence of adenosine 5′-diphosphate (ADP) (2 μmol/L; Thermo-Fisher, Waltham, MA, USA).

### *Ex-vivo* Co-culture Experiments With Collagen and Evaluation of Monocyte-Platelet Aggregates Before and After CD31 Ligation

The PBMCs at 0.5 × 10^6^/ml (0.1 × 10^6^/0.2 ml) were co-cultured *ex vivo* with platelets in a ratio of 1:1 in 96-well plates and were incubated at 37°C under 5% CO_2_ and 20% O_2_, in RPMI 1640 medium (LONZA, Verviers, Belgium), supplemented with 100 U penicillin, 0.1 mg/ml streptomycin, 2 mmol glutamine, and 10% FBS (Thermo-Fisher, Waltham, MA, USA). The mAb anti-CD31were added to each culture medium (1 μg/ml, clone WM59, 16-0319-82, Functional Grade; eBioscience, San Diego CA, USA) for CD31 ligation experiments. After 4 h of incubation, cells were fixed with fixation buffer (eBioscience™, San Diego, CA, USA) and were washed with sterile 1X DPBS (GIBCO, Invitrogen, Carlsband, CA, USA). Cells were then stained with an anti-CD14-ECD (clone RM052; B92391) and a CD42b-FITC (clone SZ2, IM0648U) (both Beckman Coulter, Brea, CA, USA) as monocyte and platelet markers, an anti-CD69-Allophycocyanin (APC) (clone TP1.55.3, A80711; Beckman Coulter, Brea, CA, USA) and an anti-CD62P-PE (clone Psel.KO2.3, 12-0626-82; eBioscience, INC., San Diego, CA, USA) to determine the monocyte and platelet activation. After the incubation, cells were washed with 0.5 ml of 1X DPBS (Gibco, Thermo-Fisher, Waltham, MA, USA) and were pelleted by centrifugation (250 X g for 5 min), the supernatant was discarded. Finally, cells were resuspended in 1 ml of 1X DPBS (Gibco, Thermo-Fisher, Waltham, MA, USA) for flow cytometry analysis. For each acquisition, a total of 50,000 events were captured. Flow cytometry analyses were conducted with Cytomics FC500 Analyzer (lasers: blue 488 nm, red 631 nm; serial number AH20082) (Beckman Coulter, Brea, CA, USA) and data were analyzed with Kaluza software (Beckman Coulter, Brea, CA, USA).

Moreover, to evaluate the effect of CD31 ligation on isolated monocytes and platelets, we incubated individual cell cultures in the presence or absence of *Escherichia coli*-lipopolysaccharide (1 μg/ml; LPS; Sigma-Aldrich, S. Louis, MO, USA) and collagen (1 μg/ml; Mascia-Brunelli, Milan, IT) before and after mAb anti-CD31 (1 μg/ml, clone WM59, 16-0319-82, Functional Grade; eBioscience, San Diego CA, USA). Anti-CD69-APC (clone TP1.55.3, A80711; Beckman Coulter, Brea, CA, USA) and anti-CD62P-PE (clone Psel.KO2.3; 12-0626-82, eBioscience, INC., San Diego, CA, USA) were used for checking, respectively, the monocyte and platelet activation before and after each treatment. After the incubation, cells in 96-well plates were washed with 0.2 ml of 1X DPBS (Gibco, Thermo-Fisher, Waltham, MA, USA) and were pelleted by centrifugation (250 × g for 5 min); the supernatants were discarded. Finally, cells were resuspended in 0.2 ml of 1X DPBS (Gibco, Thermo-Fisher, Waltham, MA, USA) for flow cytometry analysis using the plate injection mode.

This last flow-cytometry analysis was conducted with CyoFlex S B2-R3-V4-Y0 (serial number AD15040; Beckman Coulter, Brea, CA, USA) and the acquired data were analyzed with CytExpert software (Beckman Coulter, Brea, CA, USA).

Note that all the flow cytometry analyses were performed within 30–60 min after the blood draw for basal samples, and immediately after 16 h for *in vitro* studies of monocyte-platelet aggregates.

### Confocal Microscopy on Co-culture of Monocytes and Platelets

We performed a series of confocal microscopy image acquisition on 16-h co-cultured cells. Cells were fixed with a 10% formalin solution neutrally buffered (Sigma-Aldrich, St. Louis, MO, USA) at RT for 15 min and were permeabilized with 0.1% Triton X-100 in PBS for 15 min. Cells were then washed two times with PBS and were blocked in 0.5% bovine serum albumin (BSA) in PBS for 20 min before incubating overnight with the primary mouse monoclonal anti-CD31 antibody (1:500 clone JC/70A, AbCam, Cambridge, UK) and a mouse monoclonal anti-CD42b-FITC antibody (1:500; eBioscience, San Diego, CA, USA). The secondary antibody for the anti-CD31 was Alexa Fluor 546-conjugated goat anti-mouse IgG (1:1,000; Thermo Fisher Scientific, Waltham, MA, USA). About 4′,6-diaminophenyl-indole (DAPI) in ProLong® Gold Antifade Mountant (Thermo-Fisher, Waltham, MA, USA) was used for nucleic acid staining. Confocal images were obtained with a Nikon A1 MP confocal scanning system connected to an Eclipse T-i microscope, with an × 40 objective plus further 1 and × 3 magnification, acquired by Nis-Elements imaging software, and were analyzed by the processing Image-J/Fiji software (LOCI, University of Wisconsin-Madison, USA). The degree of co-localization was quantified using Mander's overlap coefficient (MOC). Data are presented as mean ± SEM with respect to untreated samples, from a minimum of 50 cells.

### Statistical Analysis

Variables were assessed by the Shapiro Wilk test. For normally distributed data, Student's *t*-test was performed for statistics between two groups, or a 1-way ANOVA and 1-ANOVA for repeated measures, with Bonferroni correction, were used for multiple comparisons; in presence of unequal variance, the Welch's *t*-test was used. Non-parametric data were analyzed using nonparametric tests: the Mann-Whitney U test for comparison between two groups, and the Kruskal-Wallis test followed by Dunn's multiple tests for between-group comparisons. For all the experimental assays performed, a two-tailed value of *p* ≤ 0.05 was considered statistically significant. Statistical analyses were performed with GraphPad Prism version 8.02 for Windows (GraphPad Software, La Jolla, San Diego, CA, USA) and with an SPSS software v22.0 (IBM Corporation, Armonk, New York, USA). For flow-cytometry analyses, Kaluza and CytExpert softwares (Beckman Coulter, USA) were used.

## Results

### Study Population

**Figure 1:**
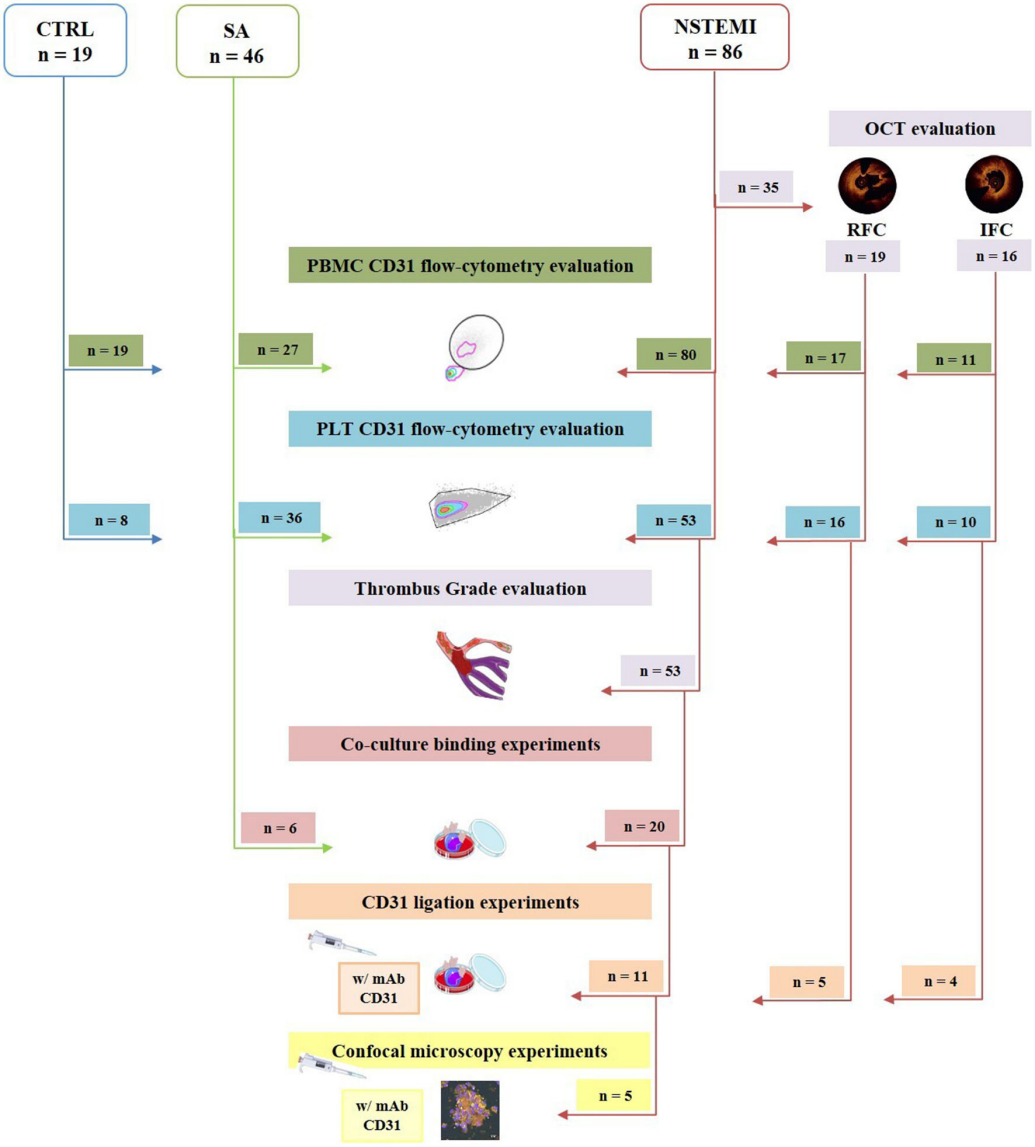
Flow chart showing the enrolled study population and its allocation within the main experimental procedures. Of 86 patients with NSTEMI, 35 underwent OCT investigation, defining *n* = 16 IFC, and *n* = 19 RFC. Obtained PBMC from 80 NSTEMI, 27 patients with SA and 19 CTRL subjects enrolled were evaluated for CD31 expression by flow cytometry. Platelet CD31 was assessed by flow-cytometry on 53 NSTEMI, 36 patients with SA, and 8 CTRL subjects. Thrombus grade analysis was performed on the latter 53 patients with NSTEMI. For co-culture binding experiments, 20 NSTEMI and 6 SA were employed. Finally, 11 were engaged for CD31 ligation experiments; of those patients, 5 were investigated through confocal microscopy. CTRL, control subjects; IFC, intact fibrous cap; NSTEMI, non-ST-elevation myocardial infarction; PBMCs, peripheral blood mononuclear cells; PLTs, platelets; RFC, ruptured fibrous cap; and SA, stable angina patients (art images from http://smart.servier.com/ site).

The main characteristics of the whole population are listed in Table 1. A flow chart describes the distribution of the enrolled population within the main experimental settings (Figure 1).

### CD31 Protein Surface Expression on Basal Monocytes and Platelets Displays a Cell-Dependent Behavior

**Figure 2:**
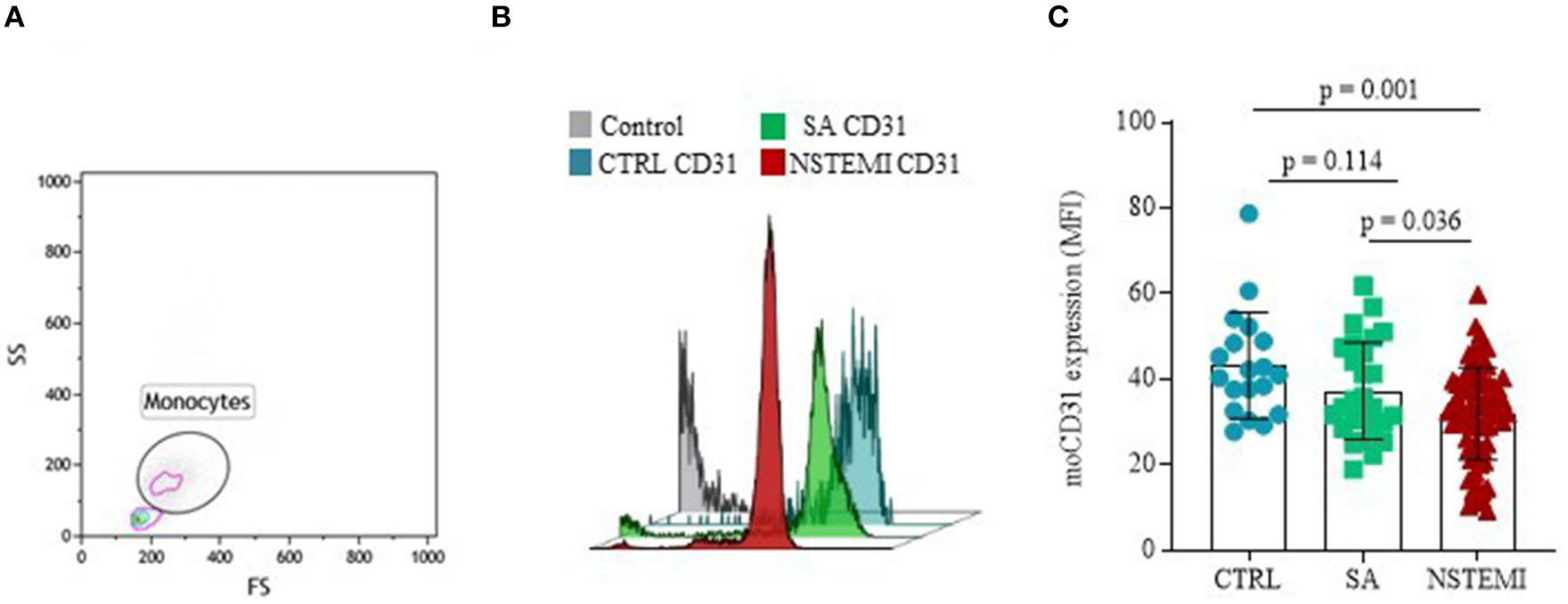
CD31 expression on monocytes. Graphs showing the surface protein expression of CD31 on CD14^+^ monocytes assessed by flow-cytometry (mean ± SD) in basal conditions. **(A)** Representative flow cytometry forward vs. side scatter plot of monocyte subset; **(B)** Representative flow cytometry overlay plot; **(C)** Data analysis of _mo_CD31 surface protein expression between the three groups (CTRL, Control individuals; FS, Forward Scatter; MFI, Median Fluorescence Intensity; _mo_CD31, monocyte CD31; NSTEMI, Non-ST-Elevation Myocardial Infarction; SA, Patients with Stable Angina; SD, Standard Deviation; and SS, Side Scatter).

To evaluate the expression of CD31 molecules on the surface of CD14^+^ monocytes and CD42b^+^ platelets, we performed multicolor flow-cytometry analyses on isolated PBMCs and platelets. We assessed the expression of CD31 on PBMCs of 80 NSTEMI, 27 SA, and 19 CTRL; meanwhile, we evaluated CD31 on platelets on 53 NSTEMI, 36 SA, and 8 CTRL subjects. The CD31 protein surface expression was significantly lower on monocytes from patients with NSTEMI (mean ± SD: 31.90 ± 10.81) compared to those from CTRL individuals (mean ± SD: 43.12 ± 12.43; *p* = 0.001) and patients with SA (mean ± SD: 37.31 ± 11.32; *p* = 0.036), respectively. No differences were observed between CTRL and SA groups *(p* = 0.114) (ANOVA for trend: *p* = 0.001) (Figure 2).

**Figure 3:**
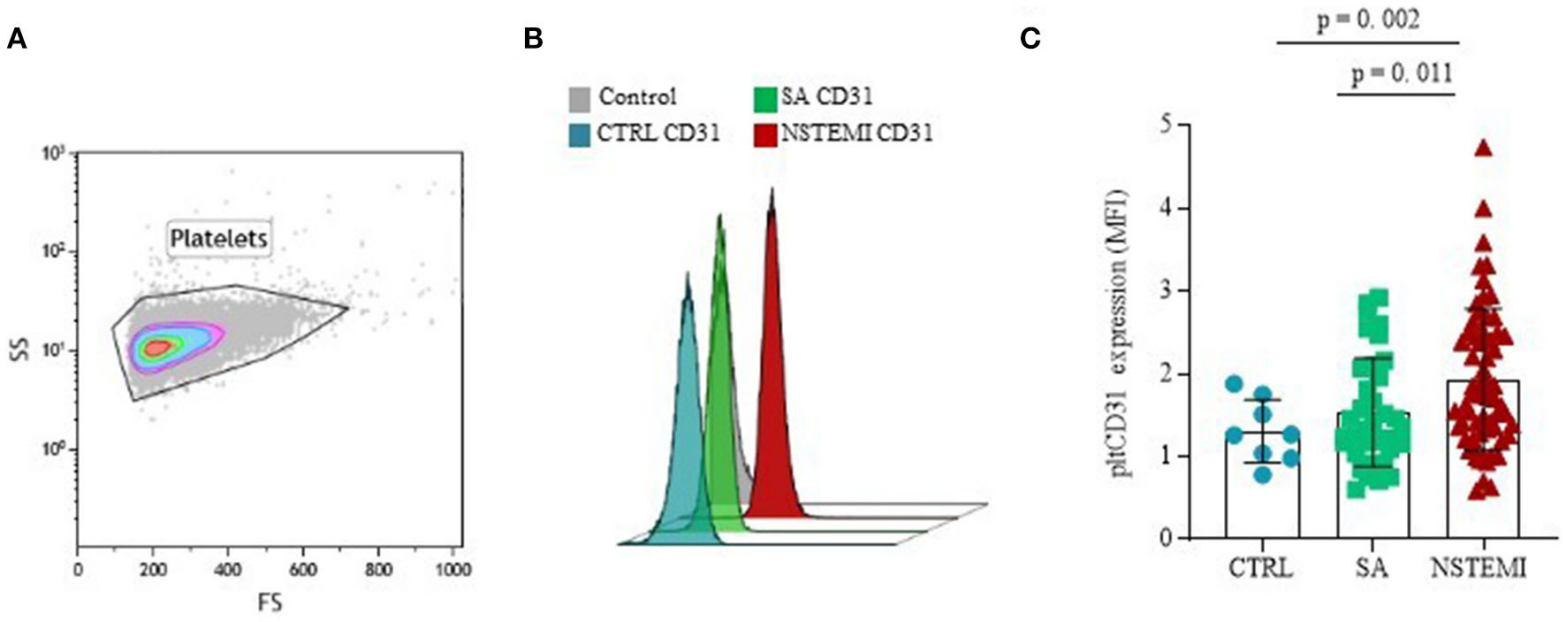
CD31 expression on platelets. Graphs showing the surface protein expression of CD31 on CD42b^+^ platelets assessed by flow-cytometry (mean ± SD), in basal conditions. **(A)** Representative flow cytometry forward *vs* side scatter plot of platelets; **(B)** Representative flow cytometry overlay plot; **(C)** Data analysis of pltCD31 surface protein expression between the three groups (CTRL, Control individuals; FS, Forward Scatter; MFI, Median Fluorescence Intensity; NSTEMI, Non-ST-Elevation Myocardial Infarction; pltCD31, platelet CD31; SA, Patients with Stable Angina; SD, Standard Deviation; and SS, Side Scatter).

In contrast, as shown in Figure 3, CD31 surface expression on basal CD42b^+^ platelets was significantly higher in NSTEMI (Mean ± SD: 1.93 ± 0.86), as compared with CTRL individuals (Mean ± SD: 1.31 ± 0.38; *p* = 0.002) and patients with SA (Mean ± SD: 1.54 ± 0.65; *p* = 0.011). No differences were observed between CTRL and SA groups (*p* = 0.209) (ANOVA for trend: *p* = 0.001). We used the P-Selectin (CD62P) as a marker of platelet activation. No difference was observed between the patients with SA and patients with NSTEMI for the CD62P expression levels (*p* = 0.942), thus, suggesting equal levels of activation of platelets in each population (Supplementary Figure 3).

### Platelet CD31 Expression Reflects a Specific Plaque Phenotype

**Figure 4:**
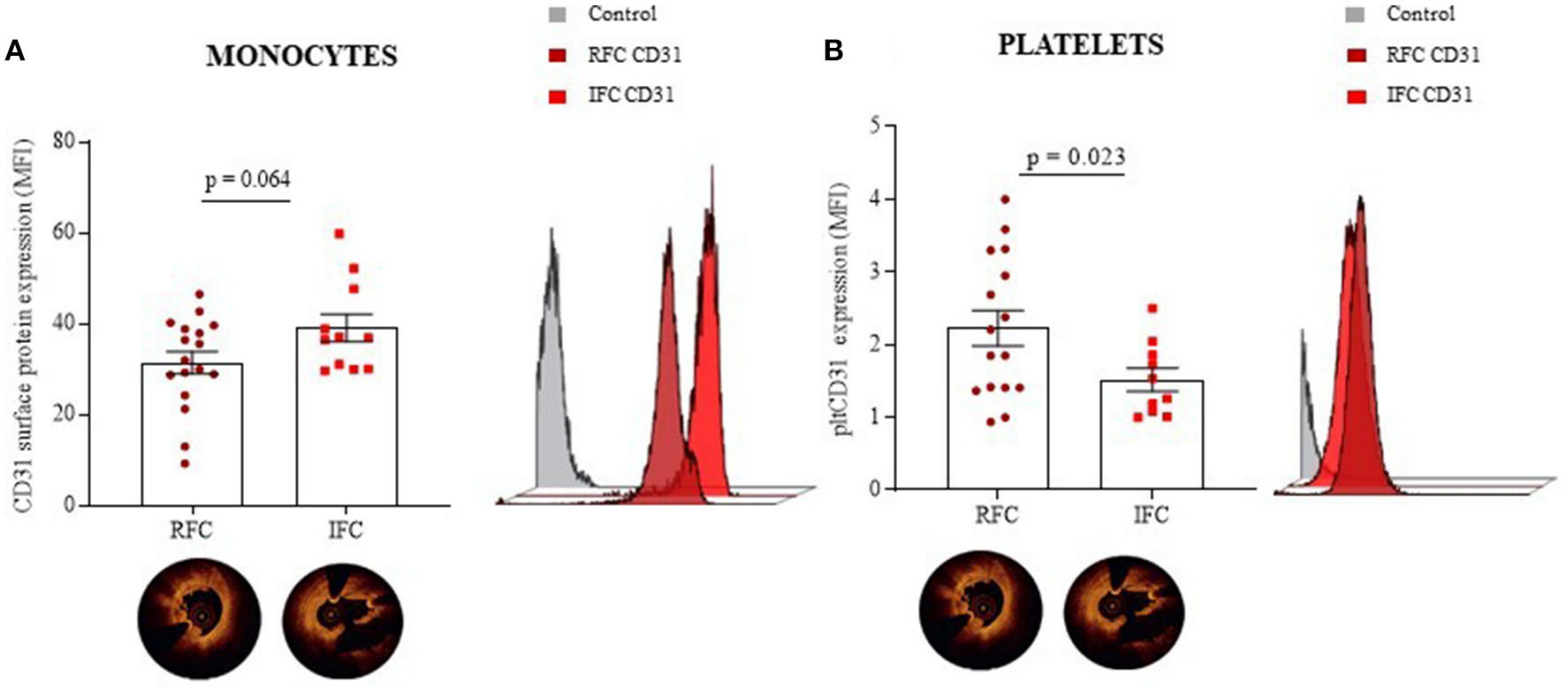
CD31 expression on monocytes and platelets according to OCT investigation. Dot plots (mean ± SD) and related representative overlay histograms showing the surface protein expression of CD31 within the NSTEMI group that underwent an OCT investigation on CD14^+^ monocytes **(A)**, and CD42b^+^ platelets assessed by flow cytometry **(B)**, in basal conditions. (NSTEMI, Non-ST-Elevation Myocardial Infarction; _mo_CD31, monocyte CD31; _plt_CD31, platelet; RFC, Ruptured Fibrous Cap; IFC, Intact Fibrous Cap; and MFI, Median Fluorescence Intensity.

From the analysis of all patients with NSTEMI who underwent intracoronary imaging, according to plaque morphology at OCT interrogation, we further sub-grouped the population of NSTEMI in RFC and IFC groups (RFC, *n* = 19; IFC, *n* = 16). Details of angiographic and OCT measurements are listed in Supplementary Table 2. The main clinical characteristics of patients with RFC and patients with IFC are presented in Table 2. No significant differences were recorded between RFC and IFC groups regarding therapies, type of dual antiplatelet therapy (DAPT), age, sex, and risk factors, except for familial history of cardiovascular disease, which is significantly higher in RFC (*p* = 0.03, see Table 2). Data revealed a decreased, although not statistically significant, expression of CD31 on CD14^+^ monocytes from patients with an RFC plaque (*n* = 17; Mean ± SD: 31.43 ± 10.15) compared to those with an IFC plaque (*n* = 11; Mean ± SD: 39.04 ± 10.04) (*p* = 0.064) (Figure 4A). In contrast, CD42b^+^ platelets from patients with an RFC plaque (*n* = 16) displayed a significant higher expression of CD31 compared to those with an IFC plaque (*n* = 10) (Mean ± SD: 2.23 ± 0.98 and 1.52 ± 0.51, respectively; *p* = 0.023) (Figure 4B). No differences were recorded between patients with NSTEMI with RFC and IFC plaques in circulating platelet numbers (*p* = 0.647) (Supplementary Figure 4).

**Table 2:** Main clinical characteristics of patients with RFC and with IFC.

	**RFC *n =* 19**	**IFC *n =* 16**	**P-value**
Age, mean ± SD	62 ± 13	63 ± 11	0.89
Gender, M/F	13/6	10/7	0.73
**CV risk factors**			
Smoke, (%)	7 (41)	10 (62)	0.20
Diabetes, (%)	5 (26)	2 (12)	0.41
Hypertension, (%)	14 (74)	11 (69)	1.00
Dyslipidemia, (%)	10 (53)	6 (37)	0.50
Obesity, (%)	4 (21)	4 (25)	1.00
Family history, (%)	10 (53)	2 (12)	**0.03**
**Medical therapy**			
DAPT, (%)^#^	6 (32)	9 (56)	0.18
ASA, (%)	12 (63)	12 (75)	0.50
Clopidogrel, (%)	3 (15)	3 (19)	0.32
Prasugrel, (%)	0 (0)	0 (0)	–
Ticagrelor, (%)	5 (26)	7 (31)	0.53
Anticoagulants, (%)	2 (10)	2 (12)	0.52
Beta-Blockers, (%)	6 (32)	9 (56)	0.24
Diuretics, (%)	4 (21)	4 (25)	0.45
ACE-I, (%)	6 (32)	4 (25)	0.33
ARBs, (%)	9 (47)	6 (38)	0.31
Statins, (%)	6 (32)	7 (43)	0.47
Ca-antagonists, (%)	2 (10)	5 (31)	0.21
Nitrates, (%)	0 (0)	1 (6)	0.46
Insulin, (%)	0 (0)	1 (6)	0.46
Oral antidiabetic, (%)	3 (16)	2 (12)	0.39

# *These data refer to the time of the enrollment of the patient and blood withdrawal. At the time of coronary angiography, all the patients with NSTEMI were on DAPT according to current guidelines. Bold values denote statistical significance. ACE-I, ACE-inhibitors; ARBs, angiotensin II receptor blockers; ASA, Aspirin; CV, cardiovascular; DAPT, dual antiplatelet therapy; IFC, intact fibrous cap; and RFC, ruptured fibrous cap*.

We further analyzed CD31 expression on monocytes and platelets from a small group of patients with STEMI, as compared to those with NSTEMI. We found no differences in CD31 expression on monocytes in the two groups (*p* = 0.118) while the CD31 expression on platelets of patients with STEMI was significantly lower as compared to those with NSTEMI (*p* = 0.0006). A gradient of CD31 expression on platelets was observed, while patients with RFC-NSTEMI showed the highest levels and patients with STEMI the lowest. These data are shown in Supplementary Figure 5.

### Platelet CD31 Expression Levels Relate to High Thrombus Burden in Patients With NSTEMI

**Figure 5:**
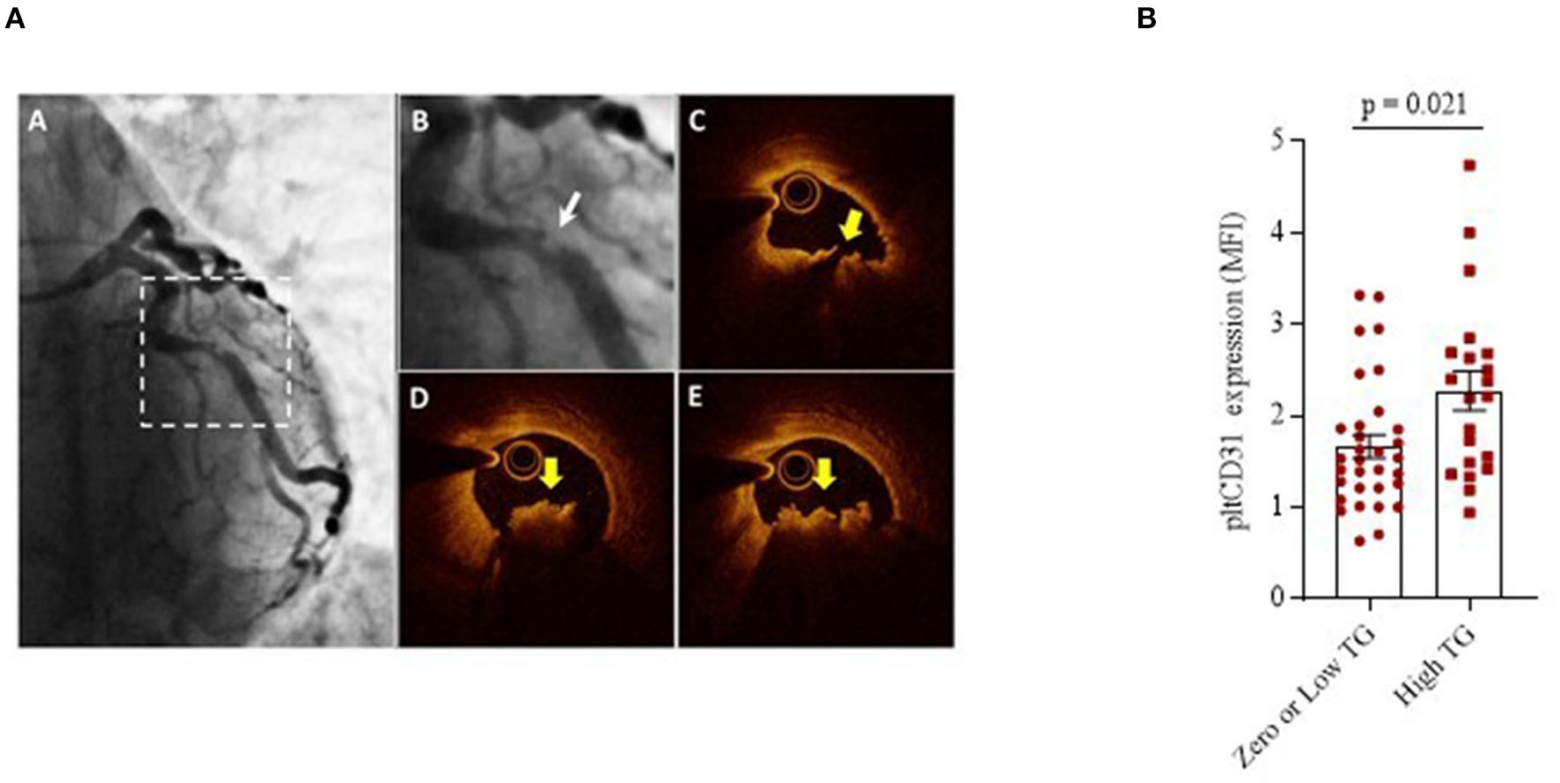
Platelet CD31 expression and Thrombus-Grade evaluation. **(A)** Representative angiographic and OCT images of a plaque. **(A)** Coronary angiogram of the left coronary artery showing a culprit lesion on the left circumflex (dotted squared selection); **(B)** Magnification of the angiogram showing a complex culprit lesion with a thrombus grade 3 and a scalloped profile (Ambrose type II eccentric); **(C)** Corresponding OCT cross-sectional image showing a lipid-rich plaque with a disrupted fibrous cap (yellow arrow); **(D,E)** Adjacent OCT cross-sections showing a large thrombus (yellow arrows). **(B)** pltCD31 surface protein expression within NSTEMI group showing the differences between patients with zero/low and high thrombus grade at the site of culprit stenosis. Data were assessed by flow-cytometry (mean ± SD), in basal conditions (MFI, Median Fluorescence Intensity; _plt_CD31, platelet CD31; SD, Standard Deviation; and TG, Thrombus Grade).

To understand whether platelet CD31 levels are related or not to thrombus burden severity at the site of the culprit stenosis, we differentiated the patients with NSTEMI (*n* = 53) according to a previously published angiographic classification, also referred to as thrombus grade score. We distinguished two groups: zero/low (*n* = 32, TG from 0 to 2) and high (*n* = 21, TG from 3 to 5) thrombus grade (Figure 5A) (42, 43). At the time of the coronary angiography, all patients were in DAPT, with no significant differences in P2Y12 inhibitor agent used as part of DAPT between zero/low and high patients with TG (clopidogrel 41.3 vs. 40%; ticagrelor 58.7 vs. 60%; prasugrel 0 vs. 0%). Results showed that the CD31 expression levels were significantly higher in platelets of patients with high TG compared to those with zero/low TG (mean ± SD: 2.27 ± 0.97 and 1.67 ± 0.71, respectively; *p* = 0.021) (Figure 5B).

### CD31 Involvement on Monocyte-Platelet Binding in Patients With NSTEMI With RFC Plaque

**Figure 6:**
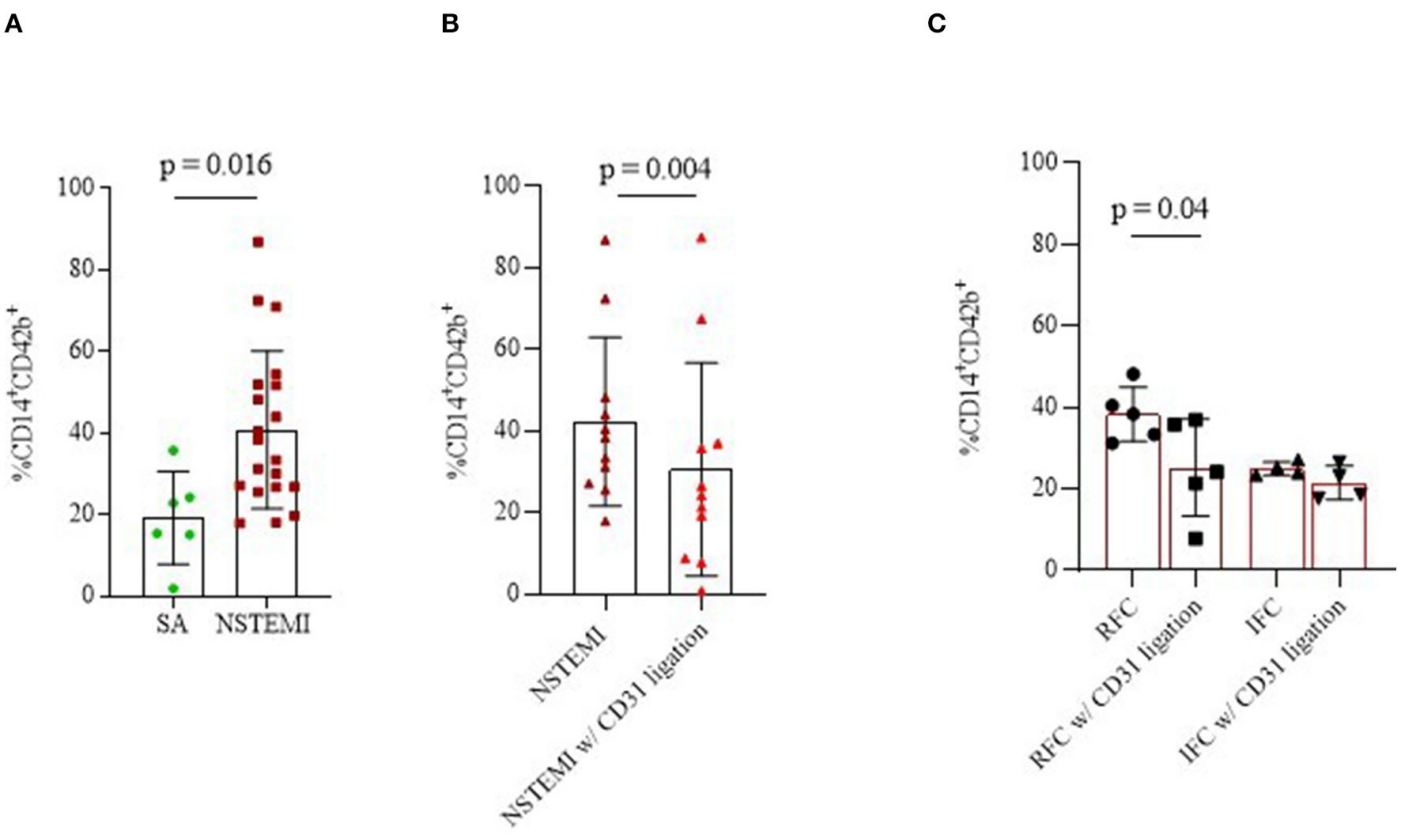
Monocyte-platelet binding. Graphs showing monocyte-platelet (Mo-Plt) binding assessed by flow-cytometry in patients with NSTEMI as compared with patients with SA **(A)**; in patients with NSTEMI before and during the CD31 ligation **(B)**, also according to an OCT investigation **(C)**. Data are presented as % of CD14/CD42b-positive cells (mean ± SD). (NSTEMI, Non-ST-Elevation Myocardial Infarction; SA, Stable Angina; SD, Standard Deviation; and w/, with).

To unravel the relationship between platelets and monocytes, we performed a series of *ex vivo* experiments by culturing them in a monolayer co-culture setting. In details, we performed 16-h co-culture experiments on cells from SA (*n* = 6) and on patients with NSTEMI (*n* = 20). As shown in Figure 6A, monocyte-platelet (Mo-Plt) binding, expressed as % of CD14-CD42b (CD14^+^CD42b^+^) positive cells, was higher in NSTEMI compared to patients with SA (mean ± SD: 40.8 ± 19.3 and 10.2 ± 11.3, respectively; *p* = 0.016). Furthermore, to test the involvement of CD31 on Mo-Plt binding, we performed the same experiment on cells isolated from patients with NSTEMI (*n* = 11) by adding mAb anti-CD31 (CD31 ligation performed with functional grade anti-Hu-CD31, clone WM59) in the co-culture medium. We observed a decreased % of CD14^+^CD42b^+^ after the CD31 ligation compared to those who were not subjected to CD31 engagement (mean ± SD: 40 ± 24 and 26.1 ± 1, respectively; *p* = 0.004) (Figure 6B). According to OCT investigation, only patients with RFC (*n* = 5) plaques displayed a decreased % of CD14^+^CD42b^+^ after the CD31 ligation compared to those who were not subjected to CD31 ligation (Mean ± SD: 38.8 ± 8.1 and 25.2 ± 12, respectively; *p* = 0.04). No effect of CD31 ligation was observed in patients with IFC (*n* = 4) (Figure 6C).

**Figure 7:**
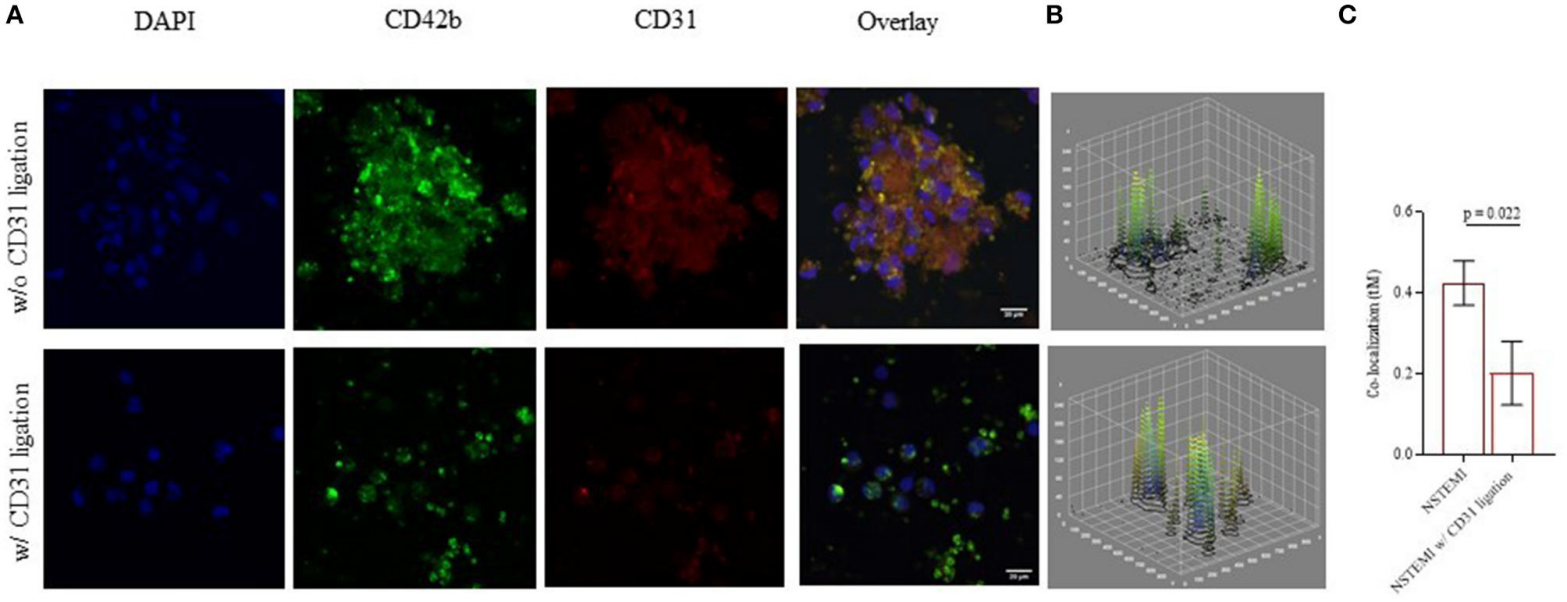
Monocyte-platelet binding at confocal microscopy. Immunofluorescence confocal microscopy images (512 × 512 pixels of resolution, scale bar 20 μm) showing monocyte-platelet (Mo-Plt) binding before and after the CD31 ligation. **(A)** A representative patient with NSTEMI; **(B)** 3D plots showing display the changing in Mo-Plt binding after the CD31 ligation; **(C)** Mo-Plt co-localization within NSTEMI group (*n* = 5). The degree of co-localization was quantified by using a Mander's overlap coefficient (MOC) (μm = micrometer; NSTEMI, Non-ST-Elevation Myocardial Infarction; w/, with, w/o, without).

Immunofluorescence confocal microscopy images and analyses within the NSTEMI group (*n* = 5) (Figures 7A,B; Supplementary Figure 6) showed a significantly reduced co-localization of mononuclear cells and platelets after the CD31 ligation (Figure 7C), confirming a flow-cytometry data (*p* = 0.022).

Additionally, to explore the involvement of platelet activation, we assessed the Mo-Plt response to adenosine diphosphate (ADP) stimulation within the NSTEMI group (*n* = 5) before and after the CD31 ligation. Data showed that CD31 ligation did not affect Mo-Plt binding of ADP treated co-cultures (*p* = 0.700) (Supplementary Figure 7).

Therefore, we set up a series of *ex vivo* co-culture experiments using samples from the newly enrolled patients with NSTEMI (*n* = 6), always before and after the CD31 ligation, in the presence or not of collagen and *Escherichia Coli*-lipopolysaccharide (LPS) for testing the effect of pro-thrombotic and pro-inflammatory stimuli on Mo-Plt aggregate formation. The CD31 ligation affects the Mo-Plt aggregate formation decreasing the % of CD14^+^CD42b^+^ on not treated (*p* = 0.003) and LPS- treated co-cultures (*p* < 0.0001) (Supplementary Figure 8A). The CD31 ligation did not affect Mo-Plt binding of collagen treated co-cultures (*p* = 0.139). We performed the same evaluation on individual cells committed in Mo-Plt binding and no differences were recorded for both CD69 and CD62P expressions, respectively, used as a marker of monocytes and platelet activation (Supplementary Figure 8B).

## Discussion

According to *post-mortem* studies, two-thirds of patients presenting with ACS show an RFC culprit plaque, rich in lipids, and inflammatory cells, as the mechanisms underpinning plaque instability, predisposing these patients to worse clinical outcomes (45). Growing evidence demonstrates that different plaque phenotypes may be the result of diverse pathogenetic mechanisms, which deserve specific therapeutic approaches (12–17). In this perspective, unusual molecular pathways at the site of the culprit plaque might beget a distinctive thrombotic burden and the antiplatelet therapy in our armamentarium might be insufficient to prevent an athero-thrombotic risk.

Our study demonstrates that circulating CD14^+^ monocytes isolated from patients with NSTEMI express lower levels of CD31 compared to patients with SA and CTRL subjects, and more interestingly, that this result is limited to patients with RFC plaques. These findings confirm the immunomodulatory role of CD31 seen in previous studies (33). Indeed, under inflammatory conditions, subsequently to the proteolytic cleavage of the N-terminal domain, leukocytes fail to express the CD31 acquiring a hyper-reactive pro-inflammatory phenotype (46). It has been widely demonstrated that the CD31-targeting antibodies block the lymphocyte transmigration restoring the immunomodulatory effects (26, 34–36). Intriguingly, in the presence of pathological wall shear stress, which is one of the eligible triggers underneath plaque rupture (47, 48), CD31 acts as a mechanosensor on endothelial cells (49–52) and might mediate a platelet adhesion on the endothelial layer and thrombosis (53).

On one hand, if the immunomodulatory role of CD31 has been established in ACS, its effects on platelet function have been poorly investigated. Platelets incubated with anti-CD31 before treatment with convulxin, a GPVI agonist, or with thrombin, have significantly decreased the intracellular Ca^2+^ mobilization levels suggesting an effect of CD31 on thrombus size and formation through platelet inactivation (32). For the first time, in our study, we noted that patients with NSTEMI under an optimal antiplatelet therapy, in particular those with RFC plaques at OCT investigation, displayed increased levels of CD31 on circulating platelets, possibly affecting the clinical outcome, as shown by the relation between the platelet CD31 expression and the angiographic thrombotic burden.

Our hypothesis has been further confirmed in mechanistic experiments showing a high Mo-Plt binding in patients with NSTEMI and with RFC, and its significant reduction after CD31-ligation. Moreover, these results seem to be independent of the exogenous over-stimulation of Mo-Plt co-cultures with common platelet activators, such as ADP and collagen. Intriguingly, CD31-ligation affects the Mo-Plt aggregate formation in LPS-treated co-cultures. These findings revealed a different mechanism of platelet activation and thrombus formation that is strongly related to the activation of inflammatory pathways, as well as to the abrupt impairment of the immunomodulatory effect of CD31 in ACS (33).

In the setting of RFC plaque, the discrepancy of CD31 expression levels between monocytes and platelets may rely on the down-regulatory activity that the CD31 exerts when it is expressed on leukocytes (33). On the other hand, on the activating role that CD31 seems to carry out on platelets (32, 36), hinting a double nature of this molecule (31). Indeed, our novel information about the *Janus*-faced CD31 expression on monocytes and platelets may suggest a differential commitment of this molecule for the different actors involved in the pathogenesis of ACS.

Our results obtained from a group of patients with STEMI confirm the multi-faced behavior and complex biology of the CD31 molecule. Although our patients in both STEMI and NSTEMI were different for the clinical settings and use of prasugrel at the time of blood withdrawal, our data are in line with the existing evidence documenting the differences in culprit plaques, the thrombus type, and the composition between patients with NSTEMI and with STEMI (54, 55).

**Figure 8:**
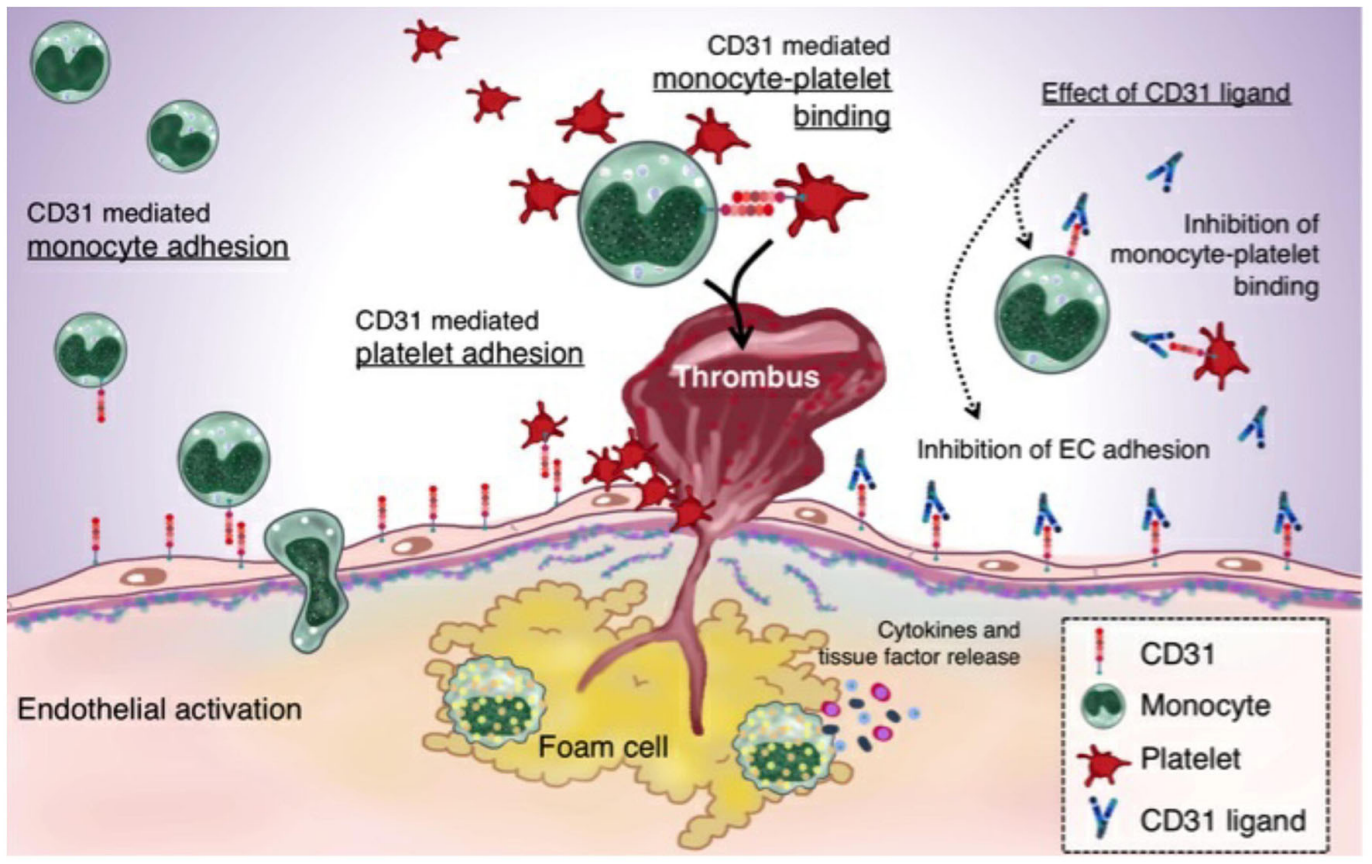
CD31 and the thromboinflammatory response of the unstable plaque: A plaque rupture paradigm. The figure summarizes the driving hypothesis that derives from both the existing evidence on plaque rupture and the data that emerged from the present study. In ACS, CD31 is involved in leukocytes and platelets adhesion on ECs. The increased monocyte-platelet (Mo-Plt) binding in the RFC *milieu* might account for an increased athero-thrombotic burden driven by the inflammation and involving, at least in part, CD31 molecule, as demonstrated by the significant reduction of Mo-Plt aggregate formation following the CD31 ligation (ACS, acute coronary syndromes; and endothelial cells, ECs).

In the era of tailored medicine, we are facing the need for further personalized therapeutic strategies (56). To this purpose, CD31 might represent a promising target (57) in the treatment of thrombotic burden (Figure 8).

### Limitations of the Study

First, population enrollment and their experimental allocation were arbitrary and mainly driven by the amount of biological material available. The OCT interrogation was performed based on clinical needs and, therefore, not all patients were included in the OCT analysis. Our study was a prospective observational analysis that included a limited number of patients. We did not include follow-up analyzes to investigate the role of the CD31 molecule during the long-term outcome. Our results obtained from a group of patients with STEMI are strongly limited by the differences in clinical management at the time of blood withdrawal and, therefore, cannot be fully compared with those obtained in patients with NSTEMI. No power calculation could be performed regarding CD31 expression on monocytes and platelets according to plaque morphology at OCT investigation because of lack of previous studies in this setting; these limitations imply that several variables other than the coronary plaque morphology might explain the observed differences across these two populations of patients with RFC and with IFC. However, no differences were found regarding clinical characteristics, therapeutic strategies including DAPT, and angiographic and OCT findings other than the type of coronary plaque morphology (i.e., RFC and IFC). Thus, CD31 might be a player in Mo-Plt aggregate formation despite the optimal therapy and DAPT, suggesting the existence of alternative thrombotic pathways, predominantly displayed in patients with NSTEMI-RFC.

Second, the use of flow cytometry as the main investigative tool should be recognized as a potential limitation of our work. Due to the limited number of cells processed, we could not proceed with parallel *ex vivo* ligation experiments using more than one anti-CD31 clone or anti-IgG1 as a reference control test. Although CD31 expression on peripheral cells has been related to local clinical features such as high thrombus grade, its involvement in the onset and progression of plaque instability relies only on its systemic assessment. In addition, the influence of DAPT therapy on thrombus grade cannot be ruled out. The presence of different pathways underpinning intracoronary thrombosis can still be argued by the observation that despite the lower CD31 platelet expression and more intensive antiplatelet regimes, patients with STEMI still display a higher TG compared with both RFC- and IFC-NSTEMI groups.

Finally, since CD31 possesses multiple glycosylation sites and multiple splicing variant sites, we should consider the resulting isoforms to reveal its cell-dependent behavior. Moreover, it is obviously essential to unravel whether the CD31-ligation in the context of Mo-Plt aggregates induces an activation or an inhibition of an intracellular-signaling dependent on pltCD31.

Therefore, further experiments are needed to the candidate the CD31 as a new therapeutic target.

## Conclusions

Our findings indicate the existence of a pro-thrombotic target that may not be related to the pathways that are usually inhibited by current antiplatelet drugs and which may have relevant clinical implications in the future management of patients with ACS with plaque rupture.

## Data Availability Statement

The original contributions presented in the study are included in the article/Supplementary Material, further inquiries can be directed to the corresponding author.

## Ethics Statement

The studies involving human participants were reviewed and approved by Ethics Committee of the Fondazione Policlinico Universitario Agostino Gemelli IRCCS—Università Cattolica del Sacro Cuore. The patients/participants provided their written informed consent to participate in this study.

## Author Contributions

RV, DP, and GL: contributed to conceptualization. RV, Ad'A, AB, EP, MP, AS, PC, FCa, GR, MD, SF, DF, LS, RP, CC, and FCri: contributed to methodology. RV, DP, GL, Ad'A, GR, RV, and RM: contributed to the investigation. GL: contributed to funding acquisition and contributed to project administration. GL, FCre, and MM: contributed to supervision. RV, DP, and GL: contributed to writing the original draft. RV, DP, GL, and FCre: contributed to writing review and editing. All authors contributed to the article and approved the submitted version.

## Funding

The present study was supported by the Italian National Project Grant PRIN 2017, Protocol 2017WJBKKW_001.

## Conflict of Interest

The authors declare that the research was conducted in the absence of any commercial or financial relationships that could be construed as a potential conflict of interest.

## Publisher's Note

All claims expressed in this article are solely those of the authors and do not necessarily represent those of their affiliated organizations, or those of the publisher, the editors and the reviewers. Any product that may be evaluated in this article, or claim that may be made by its manufacturer, is not guaranteed or endorsed by the publisher.
