# Supplementary material for: Monocyte-Platelet Aggregates Triggered by CD31 Molecule in Non-ST Elevation Myocardial Infarction: Clinical Implications in Plaque Rupture
Source: Front Cardiovasc Med. 2022 Jan 25;8:741221. doi: 10.3389/fcvm.2021.741221 (PMC8821091; doi:10.3389/fcvm.2021.741221)
Supplement: Supplementary file 1 [file Data_Sheet_1.docx]

Supplementary Materials

**Supplementary Figure 1. Flow cytometry analyses of monocytes and platelets**

**(I)**

**
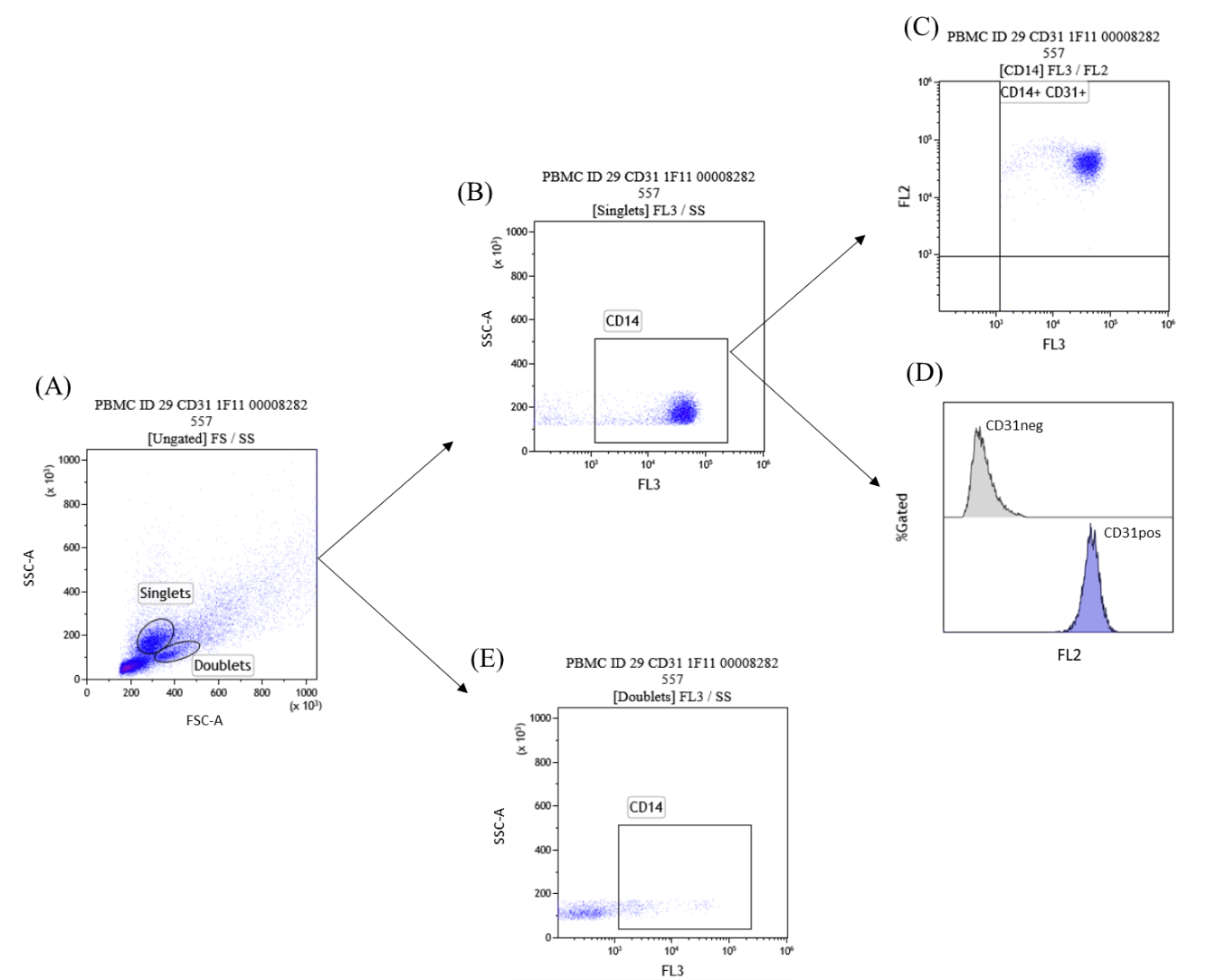
**

**(II)**


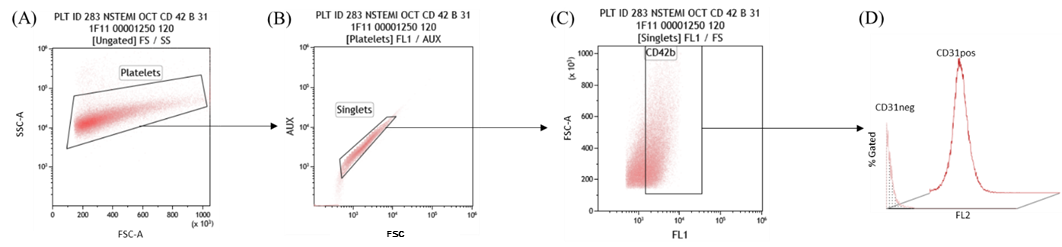


**Panel (I):** Representative example of the gating and analysis strategy of flow cytometry analyses on monocytes. **(A)** Forward *versus* Side Scatter-Area dot plots with all events is displayed (Discriminator FS, 100, flow rate 60μλ/min). A gate is created around the singlet monocyte population. **(B)** Dot plot originated from singlet gate displays monocytes with anti-CD14 antibody conjugated with ECD in FL3 and Side Scatter-Area. **(C)** Dot plot gated from CD14 and CD31 positive population showing. **(D)** Histogram offset showing CD31 positive cells *versus* control sample. **(E)** Dot plot showing doublet positive for CD14 excluded from the analysis.

**Panel (II):** Representative example of the gating and analysis strategy of flow cytometry analyses on platelets. **(A)** Forward *versus* Side Scatter-Area dot plots with all events is displayed (Discriminator FS, 100, flow rate 30μλ/min). A gate is created around the platelets population. **(B)** Dot plot originated from platelets gate displays singlets, the AUX parameter was used for doublet discrimination. **(C)** Dot plot originated from singlet gate shows platelets stained with anti-CD42b antibody conjugated with FITC in FL1 and Forward Scatter-Area. **(D)** Histogram offset showing CD31 positive cells *versus* control sample.

**Supplementary Figure 2. Flow cytometry analyses of co-cultured monocytes and platelets**


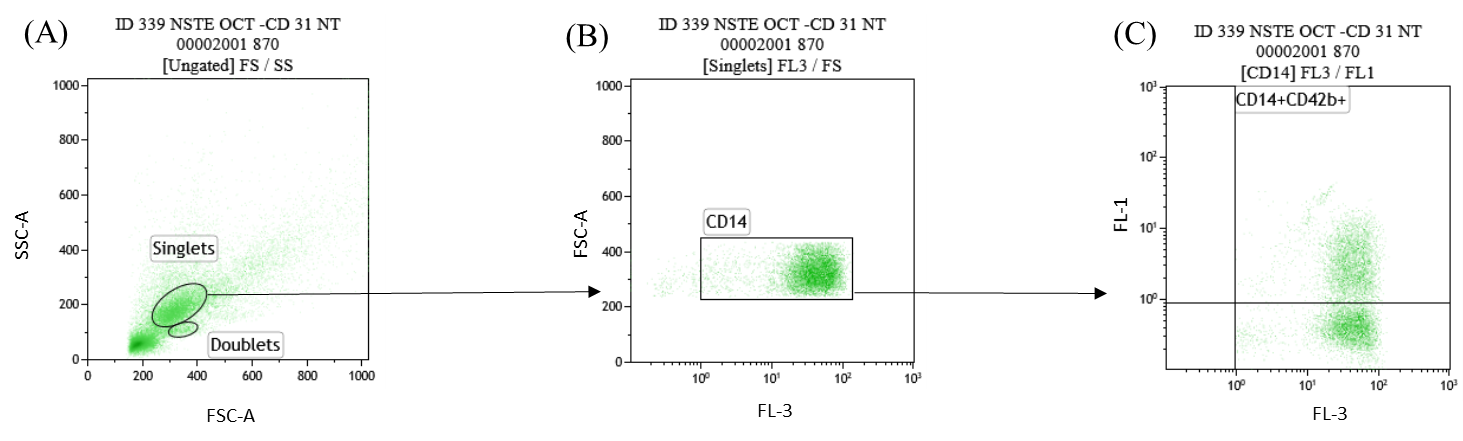


Representative example of the gating and analysis strategy of flow cytometry analyses on monocytes and platelets**. (A)** A gate is created around the monocytes, in the Forward *versus* Side Scatter dot plot with all events displayed (Discriminator FS, 100, flow rate 30μλ/min). Dot plot originated from singlet gate displays singlets. **(B)** Dot plot originated from singlet gate shows monocytes stained with anti-CD14 antibody conjugated with FITC in FL3 and Forward Scatter-Area. **(C)** Dot plot showing CD31 positive cells *versus* control sample.

**Supplementary Figure 3. CD62P expression on platelets**

Bar graph showing the surface protein expression of CD62P on CD42b^+^ platelets assessed by flow-cytometry (mean ± SD), in basal conditions, between the SA and NSTEMI patients (SA, Stable Angina Patients; NSTEMI, Non ST-Elevation Myocardial Infarction; MFI, Median Fluorescence Intensity; SD, Standard Deviation).

**Supplementary Figure 4. Circulating platelets in NSTEMI patients according to OCT investigation**

Bar graph showing numbers of circulating basal platelets assessed by Coulter counting within NSTEMI group underwent OCT (RFC, Ruptured Fibrous Cap; IFC, Intact Fibrous Cap).

**Supplementary Figure 5. CD31 expression on monocytes and platelets of STEMI patients**


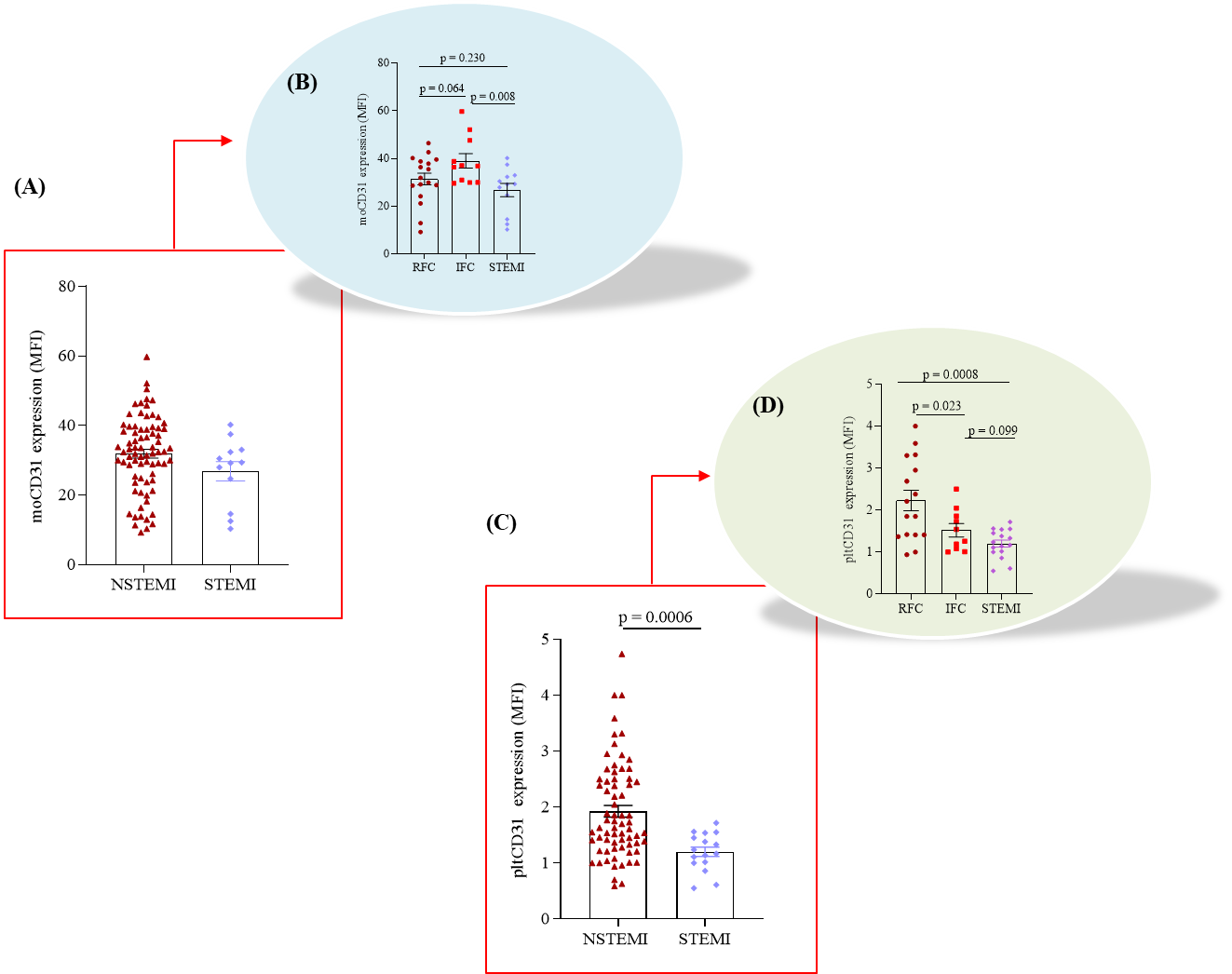


Dot plots (mean ± SD) showing statistical comparisons of CD31 surface protein expression, as assessed by flow-cytometry, between the NSTEMI and STEMI groups. There were no differences between NSTEMI and STEMI patients in CD31 expression on CD14^+^ monocytes (p = 0.118) **(A)**, while, a comparison according to OCT evaluation showed that STEMI patients had significantly lower moCD31 levels than NSTEMI patients with IFC plaques (Mean ± SD: 26.85 ± 9.66 and 39.04 ± 10.04, respectively; p = 0.008) **(B).** The same analyses performed on CD42b^+^ platelets described that STEMI patients displayed lower pltCD31 levels than the total group of NSTEMI patients (Mean ± SD: 1.20 ± 0.34 and 1.93 ± 0.86, respectively; p = 0.0006) **(C)**, and in particular STEMI patients had lower pltCD31 in comparison to the RFC-NSTEMI group (Mean ± SD: 1.20 ± 0.34 and 2.23 ± 0.98, respectively; p = 0.0008) **(D).** Supplementary Table 2 shows STEMI clinical characteristics and comparison with IFC- and RFC-NSTEMI patients. NSTEMI, Non ST-Elevation Myocardial Infarction; STEMI, ST-Elevation Myocardial Infarction; moCD31, monocyte CD31; pltCD31, platelet; RFC, Ruptured Fibrous Cap; IFC, Intact Fibrous Cap; MFI, Median Fluorescence Intensity.

**Supplementary Figure 6. Monocyte-platelet binding at confocal microscopy
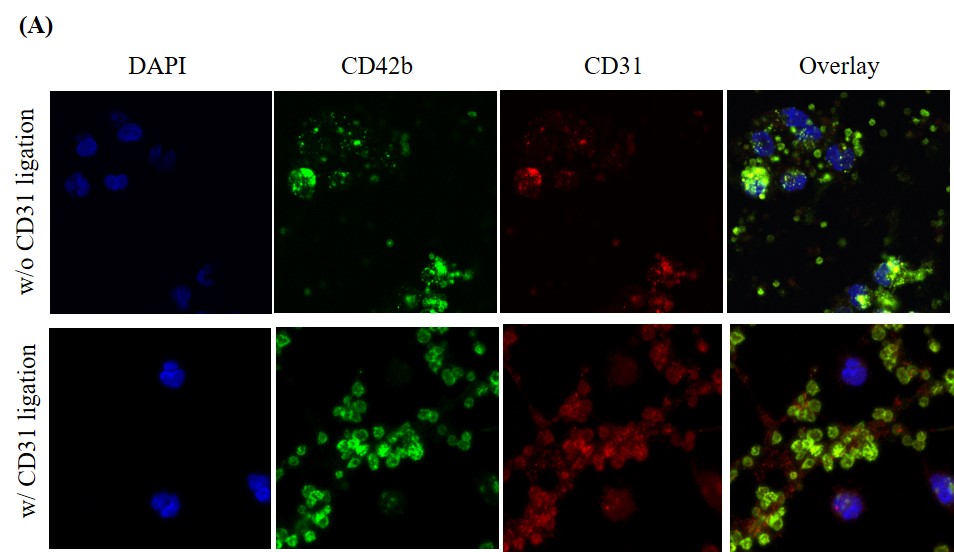
**


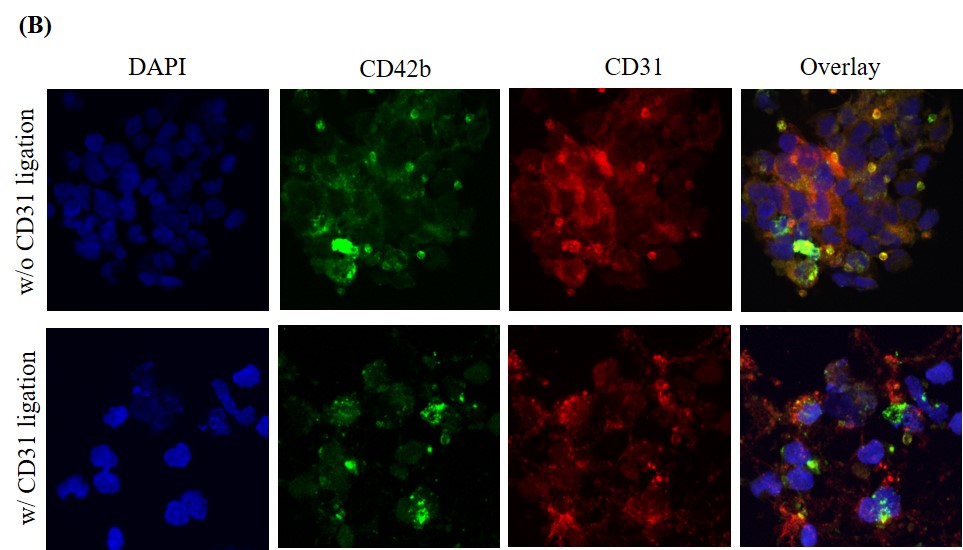


Immunofluorescence confocal microscopy images (512 x 512 pixels of resolution) of two independent experiments showing monocyte-platelet binding before and after CD31 ligation.

**Supplementary Figure 7. Monocyte-platelet binding following ADP treatment.**

**
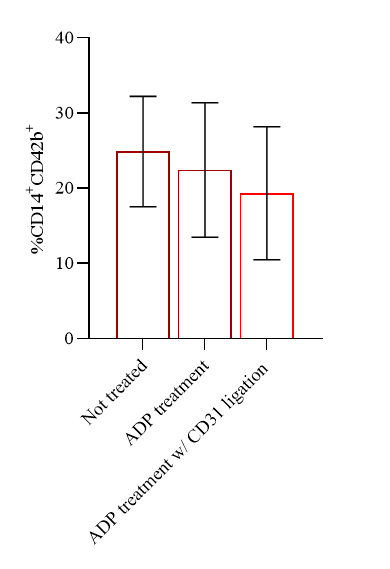
**

Bar graph showing platelet–monocyte binding assessed by flow-cytometry with or without CD31 ligation and in presence or absence of ADP. Data are presented as % of CD14/CD42b-positive cells and assessed by flow-cytometry (mean ± SD) (NT, not treated; ADP, Adenosine-5-diphosphate; w/, with; SD, Standard Deviation).

**Supplementary Figure 8.** **Effect of CD31 ligation on monocyte-platelet binding before and after treatment with collagen and LPS.**

**
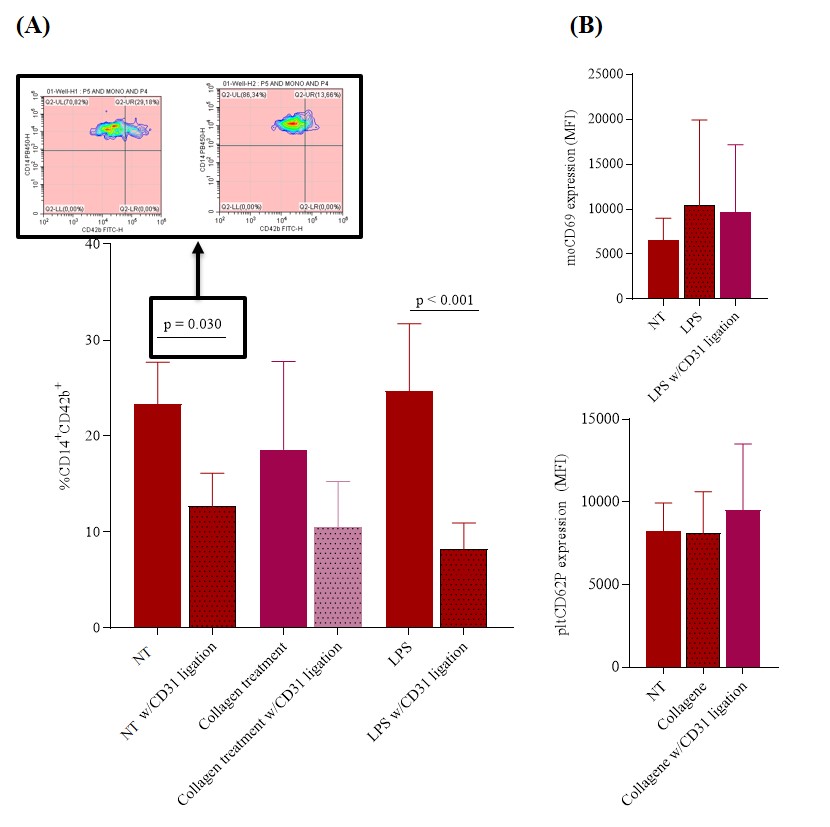
**

Bar graphs showing data from *ex vivo* experiments before and after CD31 ligation. (**A**) monocyte-platelet binding expressed as % of CD14/CD42b-positive cells in presence or not of collagen and LPS; (**B**) CD69 on *co-cultured* monocytes in presence or not of LPS and CD62P on *co-cultured* platelets in presence or not of collagen (LPS, Escherichia Coli-lipopolysaccharide _mo_CD31, monocyte CD31; MFI, Median Fluorescence; _mo_CD69, monocyte CD69; _plt_CD62P, platelet CD31; SD, Standard Deviation; w/, with).

**Supplementary Table 1. Main STEMI group characteristics and comparison with IFC and RFC.**

|  | **STEMI**  **n=16** | **RFC**  **n=19** | **IFC**  **n=16** | **pValue** |
| --- | --- | --- | --- | --- |
| Age, mean±SD | 65±12 | 62±13 | 63±11 | 0.718 |
| Gender, M/ F | 14 /2 | 13/6 | 10/7 | 0.147 |
| BMI, median (IQR) | 28 (23-30) | 28 (25-30) | 28 (24-32) | 0.930 |
| **CV Risk Factors** | |  |  |  |
| Smoke, (%) | 11 (68,7) | 7 (41) | 10 (62) | 0.137 |
| Diabetes, (%) | 3 (18,7) | 5 (26) | 2 (12) | 0.618 |
| Hypertension, (%) | 9 (56,2) | 14 (74) | 11 (69) | 0.575 |
| Dyslipidemia, (%) | 10 (62,5) | 10 (53) | 6 (37) | 0.388 |
| Obesity, (%) | 3 (18,7) | 4 (21) | 4 (25) | 0.910 |
| Family history, (%) | 1 (6.2) | 10 (53) | 2 (12) | **0.005** |
| Previous ACS, (%) | 1 (6.2) | 1 (5.2) | 3 (20) | 0.421 |
| **Angiographic parameters and post-procedure LVEF** | | | | |
| LAD culprit, (%) | 4 (25) | 6 (31.6) | 7 (43.7) | 0.496 |
| CX culprit, (%) | 4 (25) | 4 (21) | 5 (31.2) | 0.919 |
| RCA culprit, (%) | 8 (50) | 9 (47.3) | 4 (25) | 0.310 |
| Multivessel disease, (%) | 9 (56.2) | 7 (36.8) | 9 (56.2) | 0.414 |
| High Thrombus Grade, (%) | 15 (93.7) | 8 (42) | 5 (31) | **0.001** |
| LVEF >50%, (%) | 9 (56.2) | 18 (94.7) | 15 (93.7) | **0.006** |
| **Medical Therapy** |  |  |  |  |
| DAPT, (%)^#^ | 14 (87,5) | 6 (32) | 9 (56) | **0.001** |
| ASA, (%) | 15 (93,7) | 12 (63) | 12 (75) | 0.109 |
| Clopidogrel, (%) | 4 (25) | 3 (15) | 3 (19) | 0.905 |
| Prasugrel, (%) | 5 (31.2) | 0 (0) | 0 (0) | **0.004** |
| Ticagrelor, (%) | 5 (31.2) | 5 (26) | 7 (31) | 0.575 |
| Anticoagulants, (%) | 3 (18,7) | 2 (10) | 2 (12) | 0.879 |
| Beta-Blockers, (%) | 10 (62,5) | 6 (32) | 9 (56) | 0.140 |
| Diuretics, (%) | 2 (12,5) | 4 (21) | 4 (25) | 0.750 |
| ACE-I, (%) | 9 (56,2) | 6 (32) | 4 (25) | 0.153 |
| ARBs, (%) | 2 (12,5) | 9 (47) | 6 (38) | 0.085 |
| Statins, (%) | 14 (87.5) | 6 (32) | 7 (43) | **0.003** |
| Ca-antagonists, (%) | 3 (18,7) | 2 (10) | 5 (31) | 0.372 |
| Nitrates, (%) | 0 (0) | 0 (0) | 1 (6) | 0.627 |
| Insulin, (%) | 1 (6,2) | 0 (0) | 1 (6) | 0.523 |
| Oral antidiabetic, (%) | 3 (18,7) | 3 (16) | 2 (12) | 1.000 |
| **Laboratory assays (median _(IQR)_ )** | |  |  |  |
| Haemoglobin, g/dl | 13.8 _(11.7-14.9)_ | 13.5 _(12.9-14.4)_ | 14 _(13-15)_ | 0.588 |
| WBC | 10.3 _(8.9-12.4)_ | 7.5 _(5.3-10.8)_ | 8.6 _(7.2-10)_ | **0.030** |
| Platelets, 10^3^/ml | 221 _(189-281)_ | 216 _(200-230)_ | 198 _(180-251)_ | 0.440 |
| Lymphocyte count, 10^9^/l | 1.32 _(1-2.3)_ | 1.91 _(1-2.2)_ | 1.8 _(1.4-2.4)_ | 0.467 |
| Glycemia, mg/dl | 99 _(90-130)_ | 108 _(96-137)_ | 96 _(86-108)_ | 0.218 |
| Creatinine, mg/dl | 1 _(0.9-1.4)_ | 0.78 _(0.58-1)_ | 0.81 _(0.69-1.11)_ | 0.124 |
| Total cholesterol, mg/dl | 146 _(124-182)_ | 190 _(152-209)_ | 156 _(125-192)_ | 0.124 |
| LDL, mg/dl | 97 _(60-130)_ | 109 _(79-135)_ | 101 _(80-129)_ | 0.556 |
| HDL, mg/dl | 45 _(33-51)_ | 36 _(32-44)_ | 37 _(32-57)_ | 0.773 |
| Triglycerides | 95 _(62-131)_ | 141 _(104-217)_ | 136 _(125-159)_ | 0.244 |
| hs-CRP, mg/l | 8.6 _(8.2-28.9)_ | 3.1 _(0.9-4.6)_ | 9.2 _(2.5-16)_ | 0.150 |
| ESR, mm/h | 23 _(12-35)_ | 29 _(7-67)_ | 10 _(4-13)_ | 0.231 |

# These data refer to the time of patient enrollment and blood withdrawal.

ACE-I: ACE-inhibitors; ARBs: angiotensin II receptor blockers; ASA: aspirin; BMI: body mass index; CRP: C-reactive protein; CV: cardiovascular; CX: cirumflex artery; DAPT: dual antiplatelet therapy; ESR: erythrocyte sedimentation rate; HDL: high density lipoprotein; IQR: interquartile range; LAD: left anterior descending artery; LDL: low-density lipoprotein; LVEF: left ventricular ejection fraction; RCA: right coronary artery; SD: standard deviation, WBC: white blood cells.

**Supplementary Table 2. Angiographic and OCT findings.**

| **Angiographic and OCT parameters** | **RFC (n=19)** | **IFC (n=16)** | **P value** |
| --- | --- | --- | --- |
| TFG 3 | 17 (89.5%) | 16 (100%) | 0.490 |
| MLA, mm^2^ | 2.04 (1.39-2.9) | 2.6 (1.91-4.4) | 0.105 |
| Fibrous cap thickness, μm | 82 (61-90) | 245 (210-315) | **<0.001** |
| Lipid length, mm | 12 (7-17.7) | 12.9 (0-28.5) | 0.877 |
| Lipid arc, ° | 199 (184-257) | 142 (0-164) | 0.114 |

Data are expressed as mean and standard deviation for TFG 3 and median and interquartile range for the remaining data. IFC: intact fibrous cap; OCT: optical coherence tomography; MLA: minimal lumen area; μm: micrometer; mm: millimiter; RFC: ruptured fibrous cap; TFG: TIMI flow grade.
